# Effect of Cholesterol and Myelin Basic Protein (MBP) Content on Lipid Monolayers Mimicking the Cytoplasmic Membrane of Myelin

**DOI:** 10.3390/cells9030529

**Published:** 2020-02-25

**Authors:** Jennica Träger, Katharina Widder, Andreas Kerth, George Harauz, Dariush Hinderberger

**Affiliations:** 1 Institut für Chemie, Martin-Luther-Universität Halle-Wittenberg, Von-Danckelmann-Platz 4, 06120 Halle (Saale), Germany; jennica.traeger@chemie.uni-halle.de (J.T.); katharina.widder@chemie.uni-halle.de (K.W.); andreas.kerth@chemie.uni-halle.de (A.K.); 2 Interdisciplinary Research Center HALOmem at the Martin-Luther-Universität Halle-Wittenberg, 06120 Halle (Saale), Germany; 3 Department of Molecular and Cellular Biology, University of Guelph, Guelph, ON N1G 2W1, Canada; gharauz@uoguelph.ca

**Keywords:** intrinsically disordered proteins, myelin basic protein, multiple sclerosis, fluorescence microscopy, Langmuir monolayer, cholesterol

## Abstract

Myelin basic protein (MBP) is located in the insulating covers of nerve cells in the brain and spinal cord. By interacting with lipid membranes, it is responsible for compaction of the myelin sheath in the central nervous system, which is weakened in demyelinating diseases. The lipid composition of the myelin leaflet has a high impact on the interaction between the membrane and MBP. Cholesterol is present in the cytoplasmic leaflet with a rather high amount of 44% (mol%). In this study, the focus is on the effect of cholesterol, mainly by varying its content, on the interaction of MBP with a lipid monolayer. Therefore, Langmuir lipid monolayers mimicking the cytoplasmic membrane of myelin and monolayers with variations of cholesterol content between 0% and 100% were measured at the air/water interface with additional imaging by fluorescence microscopy. All experiments were performed with and without bovine MBP to study the dependence of the interaction of the protein with the monolayers on the cholesterol content. The native amount of 44% cholesterol in the monolayer combines optima in the order of the monolayer (presumably correlating to compaction and thermodynamic stability) and protein interaction and shows unique features in comparison to lower or higher cholesterol contents.

## 1. Introduction

Myelin basic protein (MBP) may play a decisive role in the elucidation of multiple sclerosis, particularly for the early stages in progression of this highly debilitating, demyelinating autoimmune disease. There are charge variants of MBP, usually termed along decreasing net positive charge from C1 to C8, that are formed by post-translational modifications (PTMs) such as deimination. PTMs and mutations in myelin proteins lead to neuropathies such as multiple sclerosis, which manifests itself in progressive demyelination [1]. In healthy adults, the mainly unmodified C1 variant has a net charge of +19 (at pH 7) and interacts with negatively charged lipids in the cytoplasmic membrane primarily via electrostatic interactions [2]. In severe cases of MS and in animal models such as EAE (experimental autoimmune encephalomyelitis), the modified, charge-reduced variants, in particular down to C8 (net charge of +13 at pH 7) are found in much greater abundance [3,4]. MBP is a membrane-based intrinsically disordered protein (IDP) [5,6]. IDPs in general possess a higher net charge and a lower proportion of hydrophobic amino acids in comparison to classically folded proteins [7]. They interact with a variety of binding partners and are highly flexible and extended but can also occupy more compact states, e.g., through electrostatic interactions [8,9,10,11]. MBP occurs in the central nervous system, where it acts as a connection between the individual layers of myelin around the axon. The 18.5 kDa splice isoform of MBP is the most abundant in the adult human brain. MBP adopts local secondary structures, in particular upon interacting with lipids, but has an extended conformation in aqueous solution [12].

On a more fundamental level, the exact order of events and specifics on the conformation, orientation, and self-assembly of MBP on a single membrane surface is not known. Studies have shown that the composition of the monolayer and the amount of protein within are important for the outcome and may be a good indication of the role in lipid bilayers such as myelin [13,14]. There are also many studies with model systems where the interaction between MBP and lipids has been investigated, but these have to be interpreted with caution because the model systems are often too complex by using the whole myelin [15,16,17] or too simple by using just parts of the lipid composition of the cytoplasmic leaflet [18,19,20]. In a previous study, we investigated this interaction with monolayer adsorption experiments by varying the composition of the lipid monolayer providing information about the specific role of each lipid [21]. In this first study, cholesterol was found to have a minor role in comparison to other lipids. In a subsequent study, the remarkable strong dependence of protein insertion (interacting in or close to cholesterol-rich regions) with only 3% sphingomyelin in the lipid monolayers, accompanied by differences for different charge states of MBP could be shown [22]. Having a minor impact on protein insertion (at least of MBP), the amount of cholesterol of nearly one half (44%) of the whole lipid amount seems very high in the natural composition. This raises the question of why the cholesterol content is that high (in most CNS membranes, it is under/about 20%) and how MBP–myelin interactions are dependent on this specific cholesterol content. The effect of specifically and systematically varying the cholesterol content has not been characterized before. Therefore, the interactions between a lipid monolayer with the general composition of the cytoplasmic myelin membrane with varying cholesterol content and MBP is investigated in the present study.

Cholesterol is an essential lipid in the membranes of mammalian cells. It consists of a sterol body with a polar 3β-hydroxyl group which interacts with the aqueous phase, and it has been shown to be located at the interface between the hydrophobic membrane made of phosphatidylcholine and the aqueous subphase of bilayers [23,24]. The hydroxyl group is smaller and less polar than a phospholipid head group. The largest moiety is the planar ring system with a short hydrophobic tail, which in a monolayer is oriented parallel to the hydrocarbon chains of the phospholipids. Furthermore, cholesterol has a condensing effect [25,26] based on the motional ordering of the phospholipid hydrocarbon chains. Less surface area is available for the phospholipids in a monolayer at the air–water interface, which leads to a decrease in the degrees of freedom of lipid motion [27,28]. Furthermore, cholesterol interrupts head group–head group interactions [29]. The condensing effect is thought to result in lipid rafts: small domains enriched in cholesterol, saturated phospholipids, and sphingolipids which are more tightly packed and have reduced fluidity in an otherwise fluid environment, which can be described as a coexistence of different lipid phases [30,31]. The transition between a two-phase region (immiscibility) and a single-phase region (miscibility) is pressure dependent and a function of the cholesterol content [26,32]. Multiple patterns of domains are possible. Stripes appear close to the upper miscible critical pressure, while circular domains form at lower surface pressures [26]. The used mixture of lipids (with 44% cholesterol), which is similar to the composition of the cytoplasmic leaflet of the myelin sheath, can coarsely be divided into charged and uncharged lipids. Phosphatidylserine (PS) and phosphatidylinositol (PI) belong to the charged anionic lipids and interact through electrostatic interactions with positively charged MBP [21]. The other neutral lipids, phosphatidylcholine (PC) and phosphatidylethanolamine (PE), are important for the overall interaction of the monolayer with MBP but seem not to interact electrostatically with MBP [21].

We first present the Langmuir monolayer results of myelin-like lipid monolayers with varying cholesterol content (0%, 10%, 20%, 30%, 35%, 40%, 44%, 50%, 60%, and 100% cholesterol) by keeping the molar ratio of the other lipids in the mixtures constant. In addition, we studied the air/water interface of the same samples by epifluorescence microscopy. All experiments were performed with and without bovine MBP to study the interaction of MBP with the monolayers in dependence of the cholesterol content. We demonstrate that 44% cholesterol in the monolayer combines the highest condensing effect (presumably correlating with a good lipid monolayer compaction and stability) and an optimum protein interaction. Moreover, the lipid system seems to be a minimalistic system and a robust mimic for the whole myelin in specific aspects. Further, it becomes clear that the composition of the monolayer is very important and the change of only one lipid has an effect on the behavior of the lipid monolayer and the interaction with MBP. Considering that, in demyelinating diseases and in the natural development of mammalian brains, myelin undergoes variations in lipid composition, this might be a future route for potential research.

## 2. Materials and Methods

### 2.1. Materials

The lipids porcine brain L-α-phosphatidylcholine (PC), porcine brain L-α-phosphatidylserine (PS), porcine brain L-α-phosphatidylethanolamine (PE), porcine brain sphingomyelin (SM), bovine liver L-α-phosphatidylinositol (PI), and cholesterol (ovine wool) were purchased from Avanti Polar Lipids (Alabaster, USA). Bovine myelin basic protein 18.5 kDa was purchased from Merck KGaA (Darmstadt, Germany). Buffer solution of *N*-(2-hydroxyethyl)piperazine-*N’*-ethanesulfonic acid (HEPES) and sodium chloride (both from Merck KGaA) was prepared with ultrapure water from a Milli-Q Advantage A10 (Millipore S.A.S., Molsheim Cédex, France) with a conductivity lower than 0.055 µS/cm, and it was adjusted with sodium hydroxide (Fisher Scientific, Leicestershire, UK) to pH 7.4. The chloroform used had HPLC grade and was purchased from Carl Roth GmbH & Co. KG (Karlsruhe, Germany). The fluorescent dye 1,2-Dihexadecanoyl-*sn*-glycero-3-phosphoethanol-amine-*N*-(lissamine rhodamine B sulfonyl) (Rh−DHPE) was obtained from Life Technologies GmbH (Darmstadt, Germany) and TopFluor^®^ Cholesterol was obtained from Avanti Polar Lipids (Alabaster, USA). All chemicals were used as received without further purification.

### 2.2. Sample Preparation

The lipid mixture had a composition similar to that of the cytoplasmic monolayer of myelin (cyt-monolayer; porcine brain L-α-phosphatidylcholine (PC), porcine brain L-α-phosphatidylserine (PS), porcine brain L-α-phosphatidylethanolamine (PE), porcine brain sphingomyelin (SM), and bovine liver L-α-phosphatidylinositol (PI) with a molar ratio of 11:13:27:3:2) [33,34]. Ovine wool cholesterol and all other lipids were dissolved in chloroform (HPLC grade) and stored at −20 °C. Lipid mixtures with cholesterol contents of 0%, 10%, 20%, 30%, 35%, 40%, 44%, 50%, 60%, and 100% were prepared. The remaining lipids were always adjusted to the same molar ratio, and the final total lipid concentrations of the spreading solutions varied from 0.6 to 1.4 mM. Bovine myelin basic protein 18.5 kDa (MBP) was dissolved in HEPES-NaCl-buffer (20 mM HEPES, 10 mM NaCl, pH 7.4) resulting in a stock concentration of 100 µM and stored at 4 °C. 

### 2.3. Langmuir Monolayers

For the measurements of surface pressure-area compression isotherms of lipid monolayers, with or without protein, the lipid mixture was spread on a subphase of buffer. A Langmuir Teflon-coated trough (266 × 99 × 3 mm^3^) was used, and the monolayer was spread dropwise with a Hamilton syringe. After spreading, a waiting period of 20 min was allowed for chloroform evaporation. The trough was equipped with a Wilhelmy balance (Riegler & Kirstein GmbH, Potsdam, Germany) and two symmetrically moveable barriers, which compressed the film with 2 Å^2^/(molecule·min). Before measurements, the trough had to be cleaned with Hellmanex and ultrapure water. All experiments were performed at 20 °C ± 0.1 °C. The overall lipid molar content was held constant at 27.9 nmol to start, in all experiments, in the gaseous phase of the isotherm. For protein experiments, an MBP stock solution was injected with a syringe through the equilibrated lipid monolayer at five different positions to promote its distribution in the buffer subphase. A final trough concentration of 100 nM MBP was used as introduced by Widder et al. [21]. Compression of the film with MBP was started when the surface pressure was constant.

### 2.4. Epifluorescence Microscopy 

Images of fluorescent monolayers at the air/water interface were recorded with an Axio Scope A1 Vario epifluorescence microscope (Carl Zeiss MicroImaging, Jena, Germany) while simultaneously recording the compression of the monolayer. The film balance (see above) below the microscope was mounted on an x-y-z stage (Märzhäuser, Wetzlar, Germany), which was motion controlled by a MAC5000 system (Ludl Electronic Products, Hawthorne, NY, U.S.A.). To ensure a dust-free environment and minimize the evaporation of water, the trough was enclosed by a home-built Plexiglas hood. The microscope was equipped with a compact light source HXP 120 C (mercury short arc reflector lamp), a long working distance objective (LD EC Epiplan-NEOFLUAR 50×), and a filter/beam splitter combination appropriate for the fluorescent dye, all from Carl Zeiss MicroImaging (Jena, Germany). Image data were recorded by an EMCCD camera (ImageEM C9100-13, Hamamatsu, Herrsching, Germany) and acquired by the software AxioVision (Carl Zeiss MicroImaging, Jena, Germany). All presented images show areas of individually contrast-adjusted raw data. Each lipid mixture was doped with 0.05 mol% Rh-DHPE (reducing the amount of brain PE by 0.05 mol%) and once with 1 mol% TopFluor^®^ Cholesterol. The monolayers were spread and the protein was injected as described above.

## 3. Results and Discussion

### 3.1. Compression Isotherms

Biological systems such as the cell membrane are complex to model in fundamental research. Depending on the cell type, they consist of a lipid bilayer with adsorbing or penetrating proteins. To model the cell membrane for experiments, it is often necessary to simplify the membrane system. This is possible by using a bilayer with reduced amount of different lipids in the composition. Forming monolayers (half a bilayer) in an interfacial environment [35,36] is also a common practice, because properties such as the phase state, phase transition [37], and the influence of the subphase or lipid composition [35] on proteins can be investigated, especially if they are not transmembrane spanning proteins.

To form a Langmuir monolayer film, the amphiphilic molecules (lipids) are arranged on the air/water interface of the aqueous subphase. The surface pressure π is defined as the decrease in surface tension of a subphase when a monolayer is spread and compressed at the air/water interface [37]. A Wilhelmy plate arrangement is used to measure the surface pressure, and the surface pressure itself provides information about the interaction between protein and lipid layer at the air/water interface [38,39,40]. 

In this study, all measurements were performed at 20 °C with a subphase of HEPES-NaCl buffer (with 100 nM MBP). The investigated lipid mixtures were a combination of different natural lipids similar to the cytoplasmic leaflet of myelin [33,34]. Mixtures of 0%, 10%, 20%, 30%, 35%, 40%, 44%, 50%, 60% and 100% cholesterol were measured, keeping the molar ratios of the other lipids constant (PC/PS/PE/SM/PI 11:13:27:3:2). 

**Figure 1:**
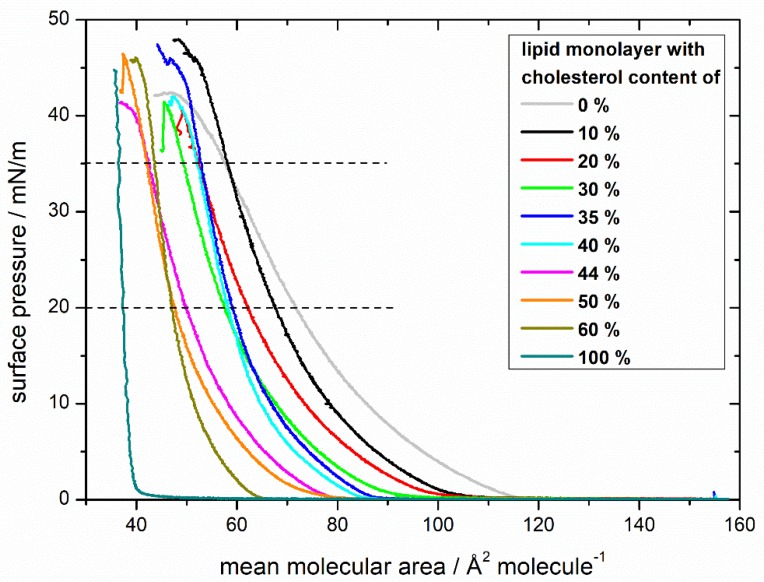
Comparison of compression isotherms of lipid monolayers with differing cholesterol contents. Monolayers have a composition similar to the cytoplasmic leaflet of the myelin sheath with varying amounts of cholesterol (0%, 10%, 20%, 30%, 35%, 40%, 44%, 50%, 60%, 100%) on a *N*-(2-hydroxyethyl)piperazine-*N’*-ethanesulfonic acid (HEPES)-NaCl subphase. The dashed lines at 20 and 35 mN/m visualize the area values used for further analysis.

As a reference, compression isotherms of lipid mixtures with different cholesterol content without MBP were recorded (Figure 1). In general, with rising cholesterol content, the isotherm “lift-off areas” are shifted to lower surface areas, indicating the build-up of a more compact monolayer. The isotherm of 100% cholesterol reproduces the results of Berring et al. [41], Demel et al. [42], and Rodriguez et al. [43] for pure cholesterol, rising steeply because of the film’s high rigidity in the condensed phase. The subsequent shift to higher surface areas with decreasing cholesterol contents shows the same trend as in the studies of Smaby et al. [44] and in the binary lipid systems of Berring et al. [41] and Kim et al. [45]. The overall shape of the curves remains similar: it goes from a gaseous phase into a liquid-expanded phase and collapses at high surface pressures. No phase transition in the liquid-expanded phase is visible. The behavior of the 44% cholesterol isotherm (the native composition) seems special, starting out similar to the isotherms with lower cholesterol content (30–35%) but ending in the surface areas of higher cholesterol content (50–60%), which means that with higher surface pressures, the monolayer of 44% cholesterol achieves a higher order. For mixtures with 20%, 44%, and 60% cholesterol, the theoretical ideal mean molecule area was calculated by the additive rule as Berring et al. [41] described (for further explanation, see Figure S1). In all three mixtures, the experimental values can be found at lower surface areas in comparison to the theoretical ones (Figure S1), showing the condensing and ordering effect of cholesterol. Furthermore, the excess free energies of mixing ∆G*_excess_* were calculated for surface pressures of 10, 20, and 35 mN/m for each monolayer (for more details, see Figure S2) [46,47,48,49]. The mostly negative ∆G*_excess_* values of the monolayers originate from attractive forces between molecules, leading to a condensation of the film [46,50]. At all three surface pressures, the ∆G*_excess_* values of monolayers with 10–40% cholesterol content are relatively close to 0 kJ/mol. At 44% cholesterol content, there is a decrease to more negative ∆G*_excess_* values which even decline with higher surface pressures. With higher cholesterol content, the excess free energy of mixing is increasing again. Therefore, monolayers with 44% cholesterol content have the highest condensing effect and the most attractive forces resulting in monolayers with the highest thermodynamic stability [51].

**Figure 2:**
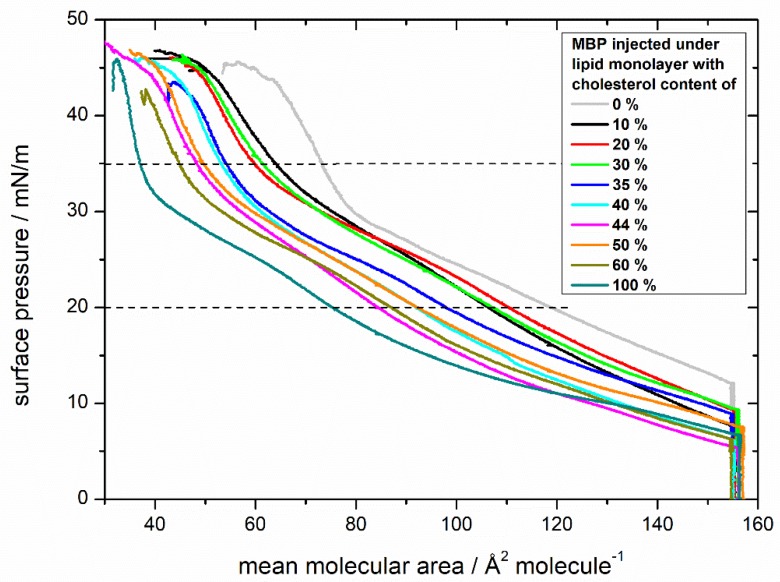
Comparison of compression isotherms of lipid monolayers with differing cholesterol contents and myelin basic protein (MBP). Monolayers have a composition similar to the cytoplasmic leaflet of the myelin sheath with varying amount of cholesterol (0%, 10%, 20%, 30%, 35%, 40%, 44%, 50%, 60%, 100%) on a HEPES-NaCl subphase. Myelin basic protein was injected into the subphase (100 nM). The dashed lines at 20 and 35 mN/m divide the isotherms in the three discussed regions and visualize the area values taken for further analysis (Figure 3).

First, experiments with MBP were performed at a surface pressure of 20 mN/m (liquid-expanded phase). The monolayer was compressed to 20 mN/m, and then MBP was injected (Figure S3a). This was done to observe the membrane pressure of 30–35 mN/m, at which the physical properties of the monolayer are similar to the corresponding bilayer [52]. After the injection of the protein under the lipid monolayer, the surface pressure rose gradually up to 27 mN/m. For comparison, MBP was also injected at 0 mN/m (gaseous phase) to study the lipid protein interaction over the whole compression isotherm range (Figure 2). It is apparent that the isotherms of both injection experiments look different, but that the fluorescence images at 25 mN/m have the same features, indicating comparable results of these different procedures (see Figure S3b). Furthermore, the experimental setup is not suitable for measurements longer than 4 hours, which is the case for injection experiments of MBP at 20 mN/m, because there is no height level-control of the subphase incorporated. The volume of the subphase decreases over time by evaporation, and it is non-negligible after approximately 4 hours in this setup. Therefore, for all the upcoming experiments, the injection of MBP in the gaseous phase was selected for further evaluation.

The isotherms of the monolayers of different cholesterol content with MBP injected below the monolayer, even at first sight, display enormous differences (Figure 2). The curves after the injection of MBP do not only lift off at a much higher surface area, but also their development strongly differs from those without MBP. The higher surface area is explainable by the incorporation of the protein into the lipid monolayer either by interacting with the acyl chain or the lipid head group [53]. The surface pressure curve can be divided into three regions as indicated by dashed horizontal lines in Figure 2. Initially, the curve rises continuously upon compression up to approximately 20 mN/m, followed by a second region from 20 mN/m until ca. 35 mN/m with a minor rise, indicating a phase transition behavior. This transition is clearly visible in the compressibility graphs for every monolayer with MBP (Figure S4). In the range between 30 and 35 mN/m, the surface pressure increases steeply until the monolayer collapses at approximately 45 mN/m. All isotherms have the same general shape. The overall behavior of the isotherms with MBP show a similar tendency as the isotherms without MBP with respect to the order of the monolayer: in both cases (Figure 1 and Figure 2) the monolayers achieve a higher lipid order with higher cholesterol content. 

**Figure 3:**
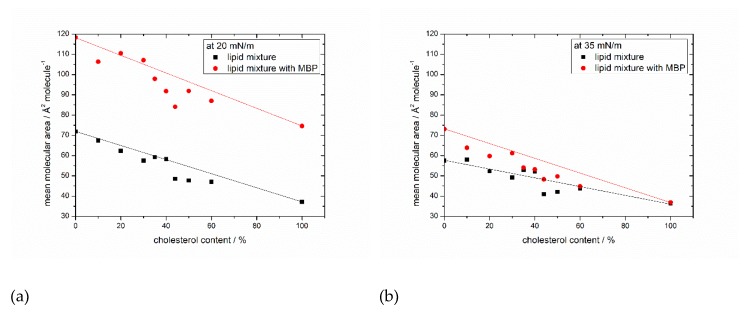
Surface area in dependency of the cholesterol content. Surface areas of lipid monolayer with (red) and without MBP (black) taken from the isotherms of Figure 1 and Figure 2 at surface pressures of (**a**) 20 mN/m and (**b**) 35 mN/m. Straight lines indicate the theoretical ideal mean molecular surface areas.

In Figure 3, the mean areas per molecule determined at pressures of 20 mN/m (Figure 3a) and 35 mM/m (Figure 3b, see also the horizontal dashed lines in Figure 1 and Figure 2) are plotted for all monolayer films without and with MBP. The general trend of the monolayers of becoming more ordered is reflected in decreasing areas per molecule with increasing cholesterol content. At 20 mN/m, the difference between the surface areas of lipids and lipids interacting with MBP on average is 41 ± 5.6 Å^2^/molecule (Figure 3a), strongly indicating that MBP interacts with the lipid monolayer, as otherwise (weak interaction) the area values should be largely identical. The difference in the surface area per molecule can be calculated to be approximately 30–36 lipids per MBP molecule (from the number of lipids for the complete monolayer by the number of proteins with a surface area of 1300 Å^2^ (on average from three α helices) at 20 mN/m). This value coincides with the findings of 36:1 by Sankaram et al. [54] by chemical binding assays and 38:1 by MacNaughtan et al. [55] by X-ray diffraction. In addition, it has been shown that the amount of positively charged amino acids (31) correlates with the deduced amount of 31 negatively charged lipids [56], which lies in the here calculated range of lipids. Furthermore, at the natural composition with 44% cholesterol with MBP, there is a clear dip in the otherwise steadily decreasing graph visible. As shown by the straight lines in Figure 3a,b which indicate the theoretical ideal mean molecular surface areas, the condensing effect of cholesterol with and without MBP is the strongest with 44% cholesterol in the monolayer. Already achieving a higher order without protein by condensation seems to be a good prerequisite for the interaction with MBP because the condensing effect gets intensified. 

From 50% cholesterol content onward, and with MBP in the film, the condensing effect weakens. A higher order may result in better access for MBP to the interaction sites as, e.g., negatively charged lipids. MBP is known to act as molecular glue, and this tightens the myelin sheath. It seems by interacting with one membrane, half of it also compacts the monolayer itself by pulling the negatively charged lipids together. This is maybe necessary to anchor (non-covalently) the α-helices in the membrane. This points out that the lipid mixture with 44% cholesterol, as also found in human cytoplasmic cells, seems to form an even more compact membrane layer (much reduced area per molecule needed), which interacts best with MBP. However, at surface pressures of 35 mM/m (Figure 3b), there is no significant difference in the surface areas that are visible, and the lipid monolayer behaves as if no proteins were present. The condensing effect is again the highest at 44% cholesterol content, but the intensification with MBP is missing. These findings combined lead to the conclusion that MBP strongly interacts with the lipid monolayer and reaches a maximum surface pressure where the interaction is most efficient. After this point, the pressure in the system gets too high and MBP probably gets squeezed out from the monolayer (it may still, e.g., adhere from the liquid side but with almost negligible contribution to the pressure). However, there is still some interaction between protein and lipids at high surface pressures above 35 mN/m. This suggestion is also supported by the results of Rosetti and coworkers [57], who have also studied the surface behavior with Langmuir films but of the whole myelin. Likewise, the maximum insertion pressure (MIP) of MBP in myelin-like monolayers is very high (42 mN/m) [21], which rather indicates a sustained attachment of MBP to the monolayer. It could be that MBP indeed orients itself in a conformation that just minimally interacts with the monolayer and that the area occupied by MBP in the interface layer is minimal. This could originate from an insertion of a shorter amino acid sequence or adhesion via electrostatic interactions at the lipid head groups of the monolayer. Looking at the differences between the surface areas of 20 and 35 mN/m with MBP in different mixtures of the same cholesterol content, it becomes apparent that with 44% cholesterol in the monolayer, the difference is the lowest (36 Å^2^/molecule, see SI Figure S5). This result can be interpreted as the monolayer with 44% cholesterol and MBP already achieving a stable self-organized state at lower surface pressures. This idea is further corroborated by the observation of the effect of further compressing the monolayer, which is also weaker in the MBP/native lipid mixture than with the other mixtures of lower or higher cholesterol contents. 

### 3.2. Epifluorescence Microscopy

To obtain a better, more microscopic scaled understanding of why lipid monolayers with 44% cholesterol deviate from the simple linear trends in Figure 3a, we investigated the air/water interface of the monolayer by epifluorescence microscopy. To this end, the same setup of the Langmuir trough was used with a fluorescence microscope on top. The lipid rhodamine-DHPE was used as the fluorescent dye. This phosphatidylethanolamine has a head group that is labeled with rhodamine and is a lipid dye that is incorporated in monolayer films, preferably in the liquid-expanded phase. It has been tested to have no significant influence on the monolayer behavior (pressure-area isotherms in the SI, Figure S6). We first discuss the images in films of varying cholesterol content without MBP; then, we analyze and discuss the changes when MBP is added.

#### 3.2.1. Varying Cholesterol Content without MBP

**Figure 4:**
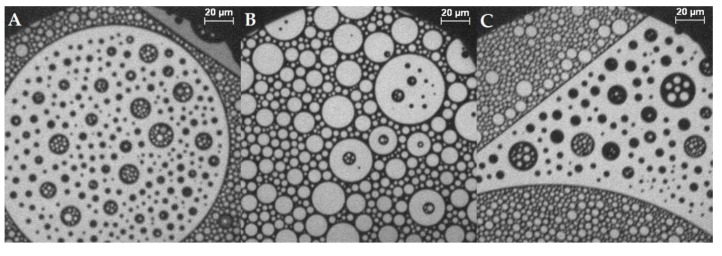

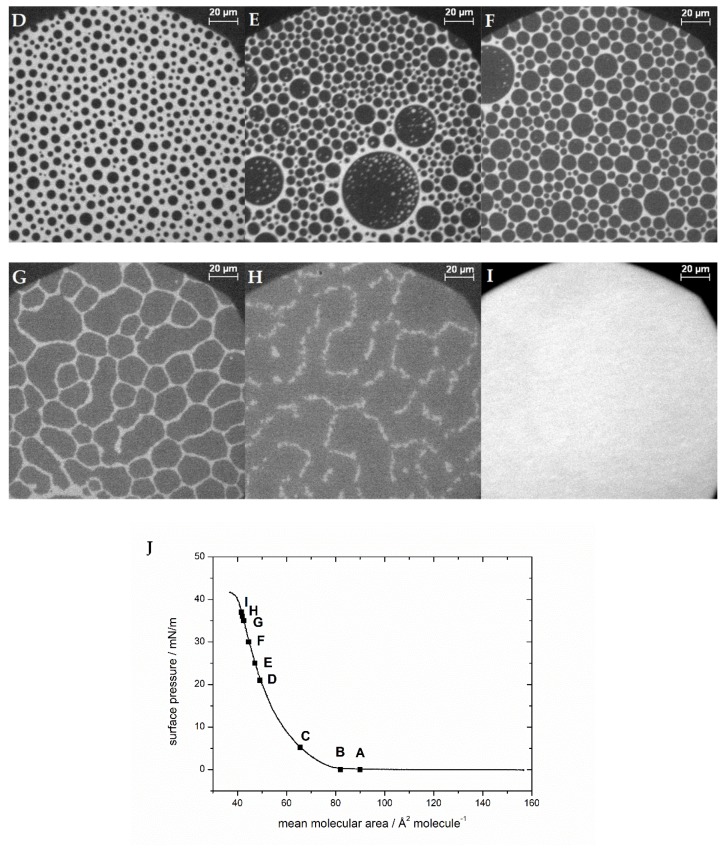
Representative fluorescence microscopy images of the compression behavior of the lipid monolayer with 44% cholesterol and 0.05 mol% 1,2-Dihexadecanoyl-*sn*-glycero-3-phosphoethanol-amine-*N*-(lissamine rhodamine B sulfonyl) (Rh-DHPE) on a HEPES–NaCl buffer subphase at ca. (**A**,**B**) 0 mN/m, (**C**) 5 mN/m, (**D**) 21 mN/m, (**E**) 25 mN/m, (**F**) 30 mN/m, (**G**) 35 mN/m, (**H**) 36 mN/m, and (**I**) 37 mN/m. The scale bar represents 20 µm. (**J**) Compression isotherm.

For monolayers without MBP, the fluorescence microscopy images show that with 44% cholesterol in the monolayer, different lipid domains are visible (Figure 4). These domains are amongst other features dark circles in the otherwise bright, liquid-expanded lipid monolayer (Figure 4D). The bright areas of the images represent the liquid-expanded phase in which the used dye, rhodamine-DHPE, partitions preferentially [58]. The dark domains are cholesterol-enriched phases that are probably liquid-ordered phases (in similarity to bilayers) or related to lipid rafts [31,59]. In a simplistic view, these model membranes consist of cholesterol and phospholipids (and sphingomyelin), and one may describe them as pseudobinary systems, as e.g., proposed by McConnell at al. [30], in which separation into a phospholipid-rich and a cholesterol-rich microphase takes place. To be sure that the dark domains are enriched in cholesterol, the same experiment was performed with a fluorescently labeled cholesterol, TopFluor^®^ Cholesterol. The result shows an inverse fluorescence image, proving that cholesterol is enriched in the dark domains (see the supporting information in Figure S7).

At cholesterol contents of 44% (shown in Figure 4) and above, the domains have a shape and form of “circles in circles” (two immiscible phases; larger isolated circular domains dispersed with smaller circular domains) (Figure 4A–C). They can be bright circles in dark ones or vice versa. The overall area of cholesterol-rich domains of monolayers with high cholesterol content is larger than that in the case of small cholesterol content. The domain size is much larger than that in samples containing low cholesterol, and domains can even be as big as the observed objective area (Ø = 240 µm). Smaller domains exhibit Brownian motion, and also, the collapse behavior changes (Figure 4B–H). First, the domains get pushed together by compressing the monolayer until there is just a minor liquid-expanded phase left between them (bright areas). The reduction in line tension leads to the circular domains, and the fact that they are spaced rather regularly may suggest repulsive interactions between them [15]. Then, the domains that had a different size distribution fuse together to form more equally sized domains (Figure 4F). When a certain size is reached, the domains do not grow any further, but they are deformed; the sharp demarcated, circular, condensed domains become less regularly shaped but are still distinctly separated. At this point, there are nearly no bright spots left in the dark condensed phase. By compressing the monolayer further, the domain borders dissolve and larger condensed areas develop. The lines of the condensed domains get fuzzy (Figure 4G), and finally the monolayer converts into one homogenously mixed monolayer. This leads to a sudden shift in fluorescence behavior, the disappearance of the condensed phase, and ending in an overall gray, homogenous image (Figure 4H,I). In other experiments, e.g., only with phospholipids, Figure 4H would correspond to a collapse of the monolayer, which is normally accompanied by a drop in the surface pressure. This is not the case here: no indication of the change in the domains is noticeable in the isotherms at pressures between 30 and 40 mN/m, at which in the films containing 44–50% cholesterol, the fluorescence images become homogenously dark first and bright upon further compression. The monolayer collapse takes place after the homogenization of the fluorescence image at surface pressures of 40–50 mN/m. 

Samples containing 50% cholesterol show the same compression behavior as those with 44% cholesterol. With 60% and 100% cholesterol, no final transition into one phase is detectable at 20 °C. The domains remain separated until the end of the isotherm is reached (SI Figure S9). We added 0.05 mol% Rh-DHPE to image the monolayer sample with (nominally) 100% cholesterol, giving a final mixture of 99.95% cholesterol and observed that PE is certainly distributed in a liquid-like phase mostly outside the black cholesterol phase, or directly at the border of the cholesterol domains. This experiment also demonstrates that a very small amount of phospholipid leads to domain formation (see Figure S8 100%) [60]. Even with only 10% cholesterol in the membrane, distinct domains are visible at the air/water interface (see fluorescence images of all samples during compression in SI Figure S9). However, these domains are just dark circles, not circles in circles. One can now follow the disappearance of the domains, when recording fluorescence images at different pressures in the isotherms, after the lift-off of the isotherm. 

Monolayers with up to 40% cholesterol without MBP show a homogeneously fluorescent interface already at low pressures (5–10 mN/m) (see Figure S8 in the SI). The first mixture studied containing more than 40% cholesterol was the sample with the native 44% cholesterol in the monolayer. From this cholesterol content onward, there is a clear change in shape and form of the domains to circles within circles, and the disappearance of the domains takes place at higher surface pressures. Furthermore, it is noteworthy that in all the monolayers, the domains form in larger patches (see fluorescence images of 44% cholesterol monolayer with a 20× magnification objective instead of 50×, Figure S10). So, the monolayer consists of areas where domains appear collectively and areas where no or fewer domains form, and the latter ones get smaller during compression of the film. McConnell et al. [26] and Keller et al. [61,62,63] observed liquid–liquid immiscibility phase diagrams for monolayers of phospholipid–cholesterol mixtures. These showed an upper critical point of immiscibility going along with the disappearance of bright stripes on a dark background. As a result of the inhomogeneous distribution of the domains and the limited observation area, it is not possible to really quantify the miscibility behavior from the fluorescence images in this study. Nonetheless, the fluorescence images of the lipid monolayers without protein also show the general trend that more cholesterol-rich domains are formed with increasing cholesterol content (see Figure S8), which withhold a higher surface pressure (end of phase separation at higher surface pressures).

#### 3.2.2. Varying Cholesterol Content with MBP

**Figure 5:**
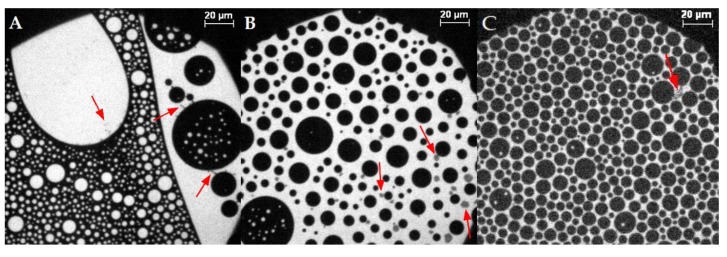

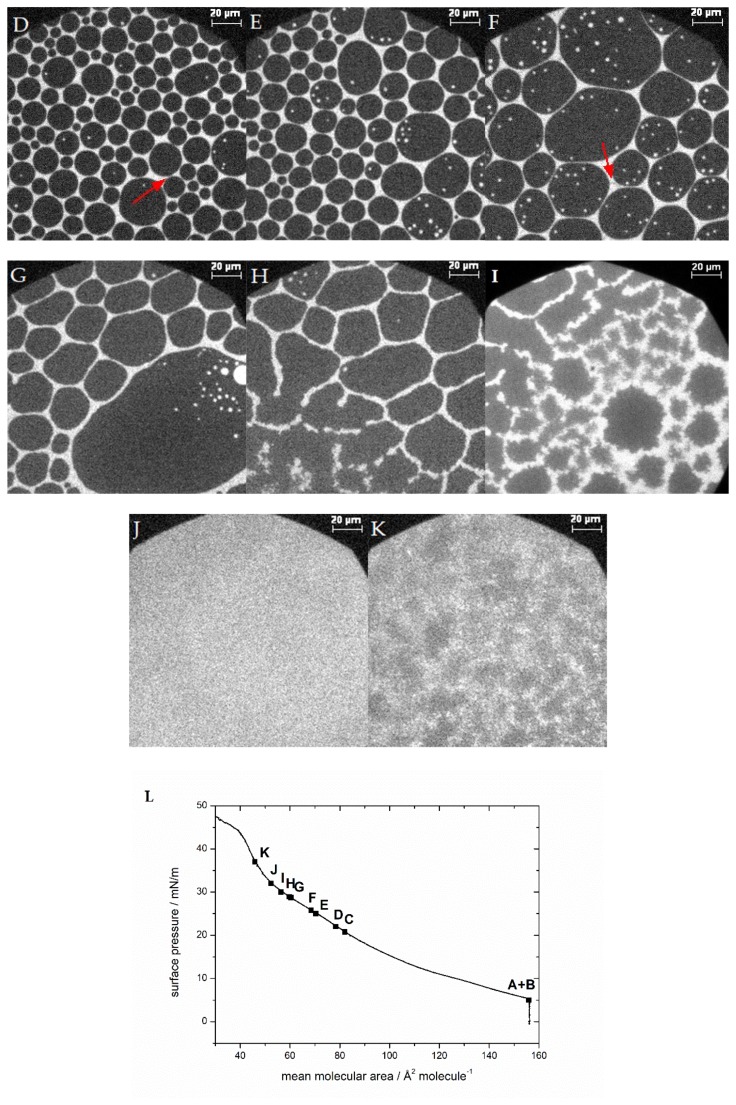
Representative fluorescence microscopy images of the compression behavior of the lipid monolayer with 44% cholesterol and 0.05 mol% Rh-DHPE on a HEPES–NaCl buffer subphase after the injection of MBP at ca. (**A**,**B**) [21] 5 mN/m, (**C**) 20.8 mN/m, (**D**) 22 mN/m, (**E**) 25 mN/m, (**F**) 25.8 mN/m, (**G**) 28.7 mN/m, (**H**) 28.9 mN/m, (**I**) 30 mN/m, (**J**) 32 mN/m, and (**K**) 37 mN/m. Red arrows highlight regions with MBP adsorption. The scale bar represents 20 µm. (**L**) Compression isotherm. A+B Reprinted with permission from Widder, K.; Träger, J.; Kerth, A.; Harauz, G.; Hinderberger, D. Interaction of Myelin Basic Protein with Myelin-like Lipid Monolayers at Air–Water Interface. *Langmuir* **2018**, *34*, 6095–6108. Copyright 2018 American Chemical Society.

With bovine myelin basic protein injected below the lipid monolayer, the domain shape of circles in circles as described above (similar to McConnell et al. [26,32]) appears in all mixtures, even with samples of lower cholesterol content than 44% (Figure 5; see comparison of all mixtures at 10 mN/m in SI, Figure S8). Mainly circular domains are formed, which is expected for liquid–liquid interfaces. The curvature of the lateral interfaces is a result of the line tension, which is described as the interfacial energy at the domain edge, and which defines the changes in growth, size, and shape of domains [64,65]. The regular spacing between mainly cholesterol-rich phases, filled with liquid-expanded phase, is due to the dipolar repulsion [32] similar to mixtures of phospholipids and cholesterol at a molar ratio of 0.3–0.4 cholesterol [32,66,67]. Moreover, the area occupied by the dark condensed phase becomes larger with higher cholesterol content in the lipid monolayer (see comparison of all mixtures at 10 mN/m in SI, Figure S8). It is interesting to note that with MBP in the subphase, gray spots appear in the microscopy images (Figure 5A,B). These spots are not visible without MBP and indicate strongly that these are interactions of MBP with the lipid monolayer [21,22]. The gray areas are always located near the condensed phase, at the interface of phospholipid-rich and cholesterol-rich domains, as described by Widder et al. [21]. The phase transition seen in all isotherms of the monolayer with MBP is in general similar to the case without protein, as described earlier for 44% cholesterol. This reflects the behavior described by Oliveira et al. [15], who also observed that a microheterogeneous surface phase of different compositions coexist in myelin monolayers made from whole myelin. They divided the topographical behavior into two sections: the first up to 20 mN/m consisting of mostly rounded and large clusters of liquid-expanded and cholesterol-rich phases, and the second describing the progressive fractal-like pattern above 20 mN/m. These authors point out that above 20 mN/m, the domains changed their form, similar to what can be observed in our study. Here, below 20 mN/m surface pressure, in the first region of the isotherm with protein, the surface pressure constantly increases while compressing the monolayer. The domains are compressed more and more (Figure 5C). In region two (see Figure 2), from 20–35 mN/m, there is a slight bulge visible in the isotherm, indicating the start of a phase transition or a protein squeeze out. This change in the phase behavior corresponds with the fluorescence images, showing the merger of domains (overcoming of repulsion) until a certain size is reached and the gray spots marking MBP disappear (Figure 5D,E). Above approximately 35 mN/m, no more gray areas are detectable. In addition, the domain borders deform from fully circular to structures with borders resembling elongated, bent lines (Figure 5F,G). With further compression, the borders get fuzzy (Figure 5H,I) as described above, which is caused by weakened line tension. The development of the microscopic images clearly indicates a reorganization of the film due to the pressure and the effect of the protein. This more detailed observation is new and was not detected before. This result substantiates the proposed “squeeze out” of MBP from the monolayers at higher surface pressures, which is in accordance with Rosetti et al. [57]. The creation of a more quantitative phase diagram of the condensed cholesterol-rich phase in dependence of the cholesterol content was not possible because of the heterogeneously distributed domains in larger patches (see SI Figure S9).

Cholesterol is well known for exerting stabilizing and compacting effects on monolayers [68,69]. This can also be observed in this study: due to a higher content of condensed domains, originating from a higher amount of cholesterol, the lipid monolayer can be compressed to lower surface areas. Cholesterol has an almost negligible impact on the electrostatic interactions with the protein [21]. If we compare the experimental results of lipid monolayers with 10% and 44% cholesterol, we find that in the former MBP incorporates itself “more favorably” into the monolayer, meaning that high surface pressures are reached at larger surface areas (Figure 2, black and magenta), and the protein interacts more favorably with the lipid monolayer. In addition, at low cholesterol content, the attractive forces of the monolayer are less, although there is more space available at the water surface. This is due to a higher amount of the charged lipids PS and PI, which have the strongest influence on MBP, resulting in electrostatic interactions [21,70,71]. This leads to lipid condensation induced by the positively charged MBP and consequently to a lower fluidity [72] of the monolayer, which results in an earlier collapse of the monolayer as well as phase transformation. The shielding influence of PE and PC [21] is negligible because the ratio of all lipids in the mixture (except cholesterol) is the same, and thus interactions between PE, PS, PC, and PI (and potentially also SM) are qualitatively and quantitatively similar. With 44% cholesterol in the monolayer, there simply is a lower share of charged lipids and thus, together with the cholesterol-rich regions, the pure electrostatic interaction between the lipid monolayer and MBP is decreased [21]. Yet, the binding/incorporation mode seems to change, as MBP, as seen in the fluorescence microscopy images and reported earlier by us (Widder et al. [21]) now seems to incorporate primarily at the border region of cholesterol- and phospholipid-rich regions. As a result of the high cholesterol content, the unoccupied, free surface for the liquid-expanded phase is decreased, but the stability (higher order, more attractive forces) of the monolayer is increased, as can be observed in pressure isotherms and fluorescence micrographs. An increase in overall compaction, even though there are more condensed domains, is due to the higher order and optimized surface area filling in the condensed phase. The liquid-expanded phase is more loosely packed, and lipids have more freedom of movement. Therefore, the acyl chains may not be aligned and on average occupy larger areas per molecule. The results of lipid monolayers with 50% and 60% cholesterol interacting with MBP show no significant improvement of monolayer stability as compared to the native content of 44%. These 44% cholesterol in the lipid monolayer seem to mark the best compromise of good interaction with MBP and fluidity on the one hand, and presumably monolayer stability on the other hand. The stability is even further enhanced when MBP is added to the monolayer, in particular, as the fluorescence microscopy images indicate that MBP addition stabilizes the cholesterol-rich regions in the lipid monolayer films.

## 4. Conclusions

Our results have shown that an amount of 44% cholesterol in the native-like lipid monolayer has a significant impact on the behavior of the compression isotherm, which is also clearly visible in fluorescence images with dye-labeled PE. In the monolayers without protein, there is a sudden appearance of ‘circles in circles’ domains, and the isotherm shows the unique behavior (in comparison to the other mixtures without MBP) that at lower surface pressures, it follows the compression trend of lower cholesterol content (30–35%), and at higher surface pressures, the surface areas are comparable to those of 50–60% cholesterol content. With higher surface pressure, the monolayer with 44% cholesterol has an increasing condensing effect, appearing to form a more thermodynamically stable monolayer. 

The overall behavior of the monolayers with and without protein is identical: with increasing cholesterol content, the surface area is decreasing because the number of cholesterol-enriched, condensed domains is increasing; therefore, the packing of the monolayer is more ordered and the surface can be further compressed. When compressing the monolayer of only a lipid mixture and thus increasing the surface pressure, domains change their shape from circular to a fuzzy state, and then to stripes, and finally to a homogeneously mixed monolayer. 

With MBP added, all mixtures with different cholesterol contents display domains of ‘circles in circles’, now in combination with gray spots. These can be identified as clusters of MBP interacting with the monolayer [21]. Furthermore, the existence of this favorable interaction is substantiated by the difference of the mean molecular area of monolayers with and without MBP at 20 mN/m (Figure 3a), showing an approximate 41 Å^2^/molecule difference. However, at 35 mN/m, the difference is almost negligible, meaning that the compression isotherms with protein at high surface pressures behave as if no protein was in the environment. This does not prohibit any interaction of MBP with lipids, but it suggests a lower impact of MBP on the ordering of the monolayer, as the MBP might change its attachment with surface pressure and may thus only be loosely bound. The fluorescence images also show the vanishing of the gray spots at higher surface pressures between 20 and 35 mN/m. Taken together, we propose a “squeeze out” in the specific pressure region where a slight bulge in the compression isotherm is visible. This means that there is a maximum pressure at which the interaction between lipids and protein is most efficient, after which the protein reorganizes in conformation and interaction to overcome the applied stress. The monolayers with 44% cholesterol content and MBP represent the most stable monolayers, and they have the biggest improvement in stability at normal membrane surface pressures of 35 mN/m in comparison to the monolayers with only phospholipids. This is a clear sign that the composition of the monolayer is very important, and just a variation of one component has a major impact on the interaction between the lipid monolayer and myelin basic protein. The 44% cholesterol mixture combines the highest condensing effect (presumably correlating with a good compaction and stability) and an optimum protein interaction. 

All in all, there clearly is an interaction between the lipid monolayer and MBP, as can be analyzed through the different effects recorded in the area-pressure isotherms with and without protein. This is reflected in the different curve behavior and the change in the fluorescence microscopy images, especially the appearance of the gray spots of aggregated MBP. It should be stated that the lipid monolayer does not necessarily represent the natural condition of the whole myelin, as it only constitutes half a bilayer. Since Oliveira et al. [15] have reported a similar behavior in whole myelin, our results show that the monolayer model we are using with just the selected lipids and MBP seems to be a robust mimic and almost minimal system for the whole myelin with respect to the overall behavior of the lipid layer and MBP interaction. As the monolayer model used is simpler and easier to handle and can be produced more accurately for changes in individual components and other proteins in future work, it has the benefit of reproducing properties seen in the whole myelin. It is still sufficiently complex so that the MBP has the basic structure of its natural environment, which is not given with a system of one or two lipids in the monolayer. Nevertheless, the monolayer model of this study should be improved to approach more physiological conditions, e.g., by adjusting salt contents [73,74]. 

As the used bovine myelin basic protein is not a pure protein sample in terms of charge and length, we therefore aim to incorporate pure recombinant forms representing the unmodified and deiminated forms of MBP (as we have done for the interaction with SM) [22] to investigate the influence of cholesterol on the healthy and diseased MBP in the monolayers. Additionally, we plan to use fluorescently labeled MBP as well. This will be the next steps for our research on the cholesterol content dependence.

## Supplementary Materials

The following are available online at https://www.mdpi.com/2073-4409/9/3/529/s1, Figure S1: Calculated ideal surface areas of lipid mixtures, Figure S2: Calculation of excess free energy of mixing, Figure S3: Comparison of different injection pressures for MBP, Figure S4: Compressibility of monolayers, Figure S5: Surface area differences of monolayers with different cholesterol content and MBP, Figure S6: Influence of fluorescent dyes on isotherm behavior, Figure S7: Fluorescence microscopy image of TopFluor^®^ Cholesterol, Figure S8: Comparison of all mixtures with and without MBP at approximately 10 mN/m, Figure S9: Fluorescence microscopy images of all lipid mixtures used, with and without MBP, Figure S10: Fluorescence microscopy images with a 20× magnification objective.
