# Supplementary material for: Effect of Cholesterol and Myelin Basic Protein (MBP) Content on Lipid Monolayers Mimicking the Cytoplasmic Membrane of Myelin
Source: Cells. 2020 Feb 25;9(3):529. doi: 10.3390/cells9030529 (PMC7140459; doi:10.3390/cells9030529)
Supplement: Supplementary file 1 [file cells-09-00529-s001.pdf]

Supporting Information

S1 Calculated ideal surface areas of lipid mixtures

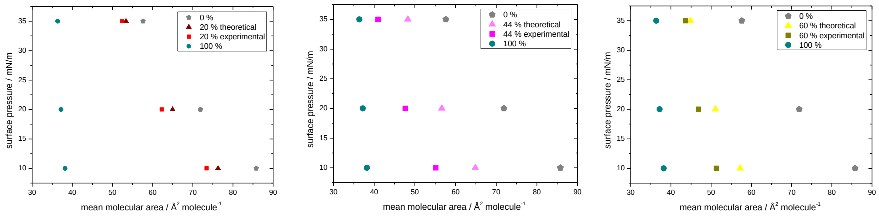

Figure S1. Experimental surface pressure-area values at 10, 20 and 35 mN/m with calculated ideal surface areas of lipid mixtures with 20 % (left), 44 % (middle) and 60 % (right) cholesterol content.

The experimental data for the surface pressure and surface area were taken from the compression isotherms at specific pressure points of 10, 20, and 30 mN/m. To calculate the theoretical values at the three surface pressures for the lipid mixtures with differing cholesterol content in the monolayer, the isotherms of just lipids (0 % cholesterol) and pure cholesterol (100 %) were taken, and in dependency of the molar ratio of cholesterol content added. Following $\bar{A} = x_{chol} * A_{chol} + x_{lipids} * A_{lipids}$ with $\bar{A}$ being the average molecular area of the monolayer, A_{chol} and A_{lipids} are the surface areas of the pure cholesterol and pure lipid isotherm, respectively. x_{chol} is the molar ratio of cholesterol in the mixture and x_{lipids} for the lipid mixture without cholesterol. The calculated average surface areas of all three cholesterol contents are located at higher surface areas in comparison to the experimental values. This demonstrates the condensing effect of cholesterol in the monolayer of lipid mixtures.

S2 Calculation of excess free energy of mixing

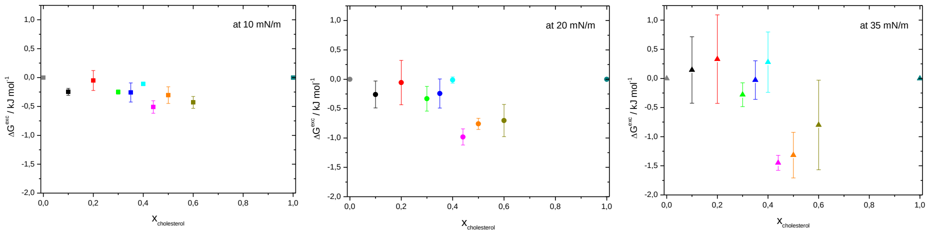

Figure S2. Calculated excess free energies of mixing ΔG^{excess} as a function of mole fraction of cholesterol $x_{cholesterol}$ from monolayers with different cholesterol content at surface pressures of 10 mN/m (left), 20 mN/m (middle) and 35 mN/m (right).

The excess free energy of mixing is calculated by $\Delta G^{excess} = \int_0^{\pi} A_{mix} - \left(x_{chol}A_{chol} + x_{lipids}A_{lipids}\right) d\pi$ for not ideal mixing. Where x_{chol} is the molar ratio of cholesterol in the mixture and x_{lipids} for the lipid mixture without cholesterol. A_{mix} is the surface area of the mixed monolayer, A_{chol} and A_{lipids} are the surface areas of the pure cholesterol and the pure

lipid isotherm, respectively. ΔG^{excess} was calculated for three different surface pressures: 10, 20, and 35 mN/m. All values for the excess free energy of mixing show a tendency to be negative or approximately 0 kJ/mol. ΔG^{excess} values are negative when the monolayer is condensing, resulting from attractive forces between unlike molecules and therefore the process of mixing is thermodynamically favored. High positive values of ΔG^{excess} suggest the immiscibility of the monolayer mixture with a possible phase separation. The excess free energy of mixing of monolayers with MBP was not calculated because the compression isotherms start at higher surface pressures which are not suitable for calculations.

S3 Comparison of different injection pressures for MBP

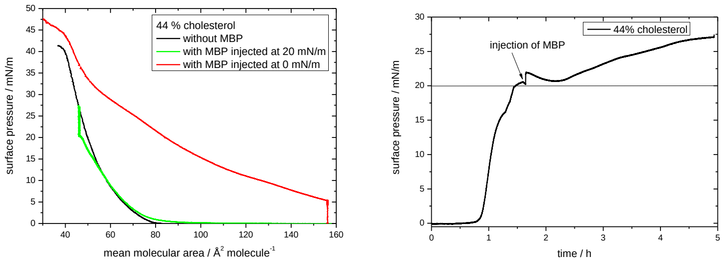

Figure S3a. Compression isotherms of 44 % cholesterol in monolayers at different injections points (left) and time-dependent surface pressure curve with injection of MBP at 20 mN/m (right).

The fast surface pressure increase followed by a slower pressure relaxation directly after the MBP injection under the lipid monolayer at 20 mN/m (Fig. 3a right) might be an experimental artifact as the protein was injected with a needle through the lipid film. After a short lag time the surface pressure gradually increased due to the MBP interaction with the lipid monolayer.

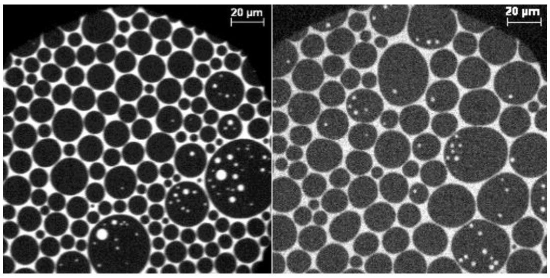

Figure S3b. Representative fluorescence microscopy image of a lipid monolayer with 44 % cholesterol at 25 mN/m with MBP injected at 20 mN/m (left) and 0 mN/m (right).

The fluorescence images in Fig. 3b depict the comparison of the two injection points of MBP at 20 and 0 mN/m at a surface pressure of approximately 25 mN/m. Both images show large domains, which are not perfectly circular anymore.

S4 Compressibility of monolayers

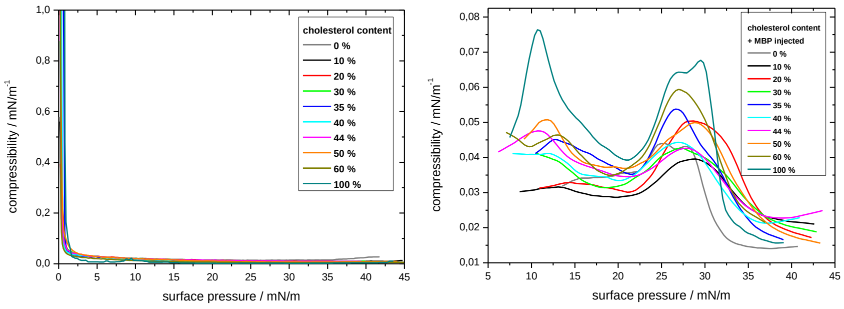

Figure S4. Compressibility-surface pressure graphs without (left) and with MBP (right).

The compressibility κ of the monolayers was calculated from the isotherms of Fig. 1 and 2 with $\kappa = -\frac{1}{A}\frac{dA}{d\pi}$. The compressibility of the monolayers with different cholesterol content shows no phase transition without MBP. But with MBP injected under the monolayers, there is a clear change in compressibility between 20 to 35 mN/m for all mixtures. This change presumably comes from the squeeze-out of the protein. The second change in compressibility at lower surface pressures (5-17 mN/m) was not examined in clearly detail. It could be a phase transition originating from cholesterol because it is most pronounced in the pure cholesterol isotherm, but could also be an artefact of the monolayer because it is a very broad transition.

S5 Surface area differences of monolayers with different cholesterol content and MBP.

Table S5. calculated surface area difference between 20 and 35 mN/m of monolayers with different cholesterol content and MBP.

Deleted: 1

Cholesterol content in %	$\Delta A = A_{20\ mN/m} - A_{35\ mN/m}$ in Å²/molecule
0	45
10	42
20	51
30	46
35	44
40	39
44	36
50	42
60	42
100	38

Listed are the differences ΔA of surface areas at 20 mN/m ($A_{20\ mN/m}$) and 35 mN/m ($A_{35\ mN/m}$) of monolayers with MBP. Values are taken from Fig. 3.

S6 Influence of fluorescent dyes on isotherm behavior

Field Code Changed

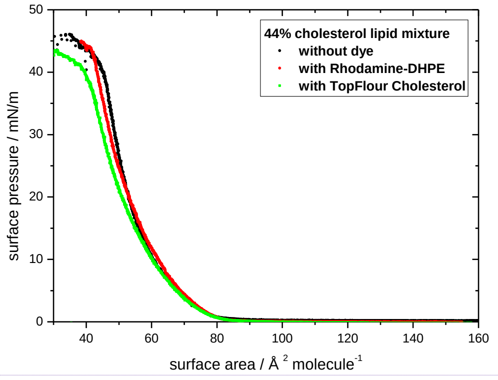

The compression isotherms of 44 % cholesterol with 0.05 mol% Rhodamine-DHPE (red) or 1 mol% TopFluor® Cholesterol (green) show no significant deviation from the 44 % cholesterol lipid monolayer without dye (black). Mixing the fluorescent dye to the lipid mixture should have a neglectable influence on the behavior of the monolayers.

S7 Fluorescence microscopy image of TopFluor® Cholesterol

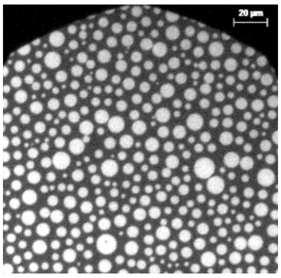

Figure S7. Representative fluorescence microscopy image of a lipid monolayer with 44 % cholesterol and 1 mol% TopFluor® Cholesterol at ca. 10 mN/m.

The bright areas are cholesterol-rich domains with the dye and the dark area is the phospholipid-rich liquid-expanded phase. The image is exactly inverted to the fluorescence

images of Rhodamine-DHPE, proving that cholesterol is, indeed, enriched in the round domains.

S8 Comparison of all mixtures with and without MBP at approx. 10 mN/m

Without MBP | with MBP

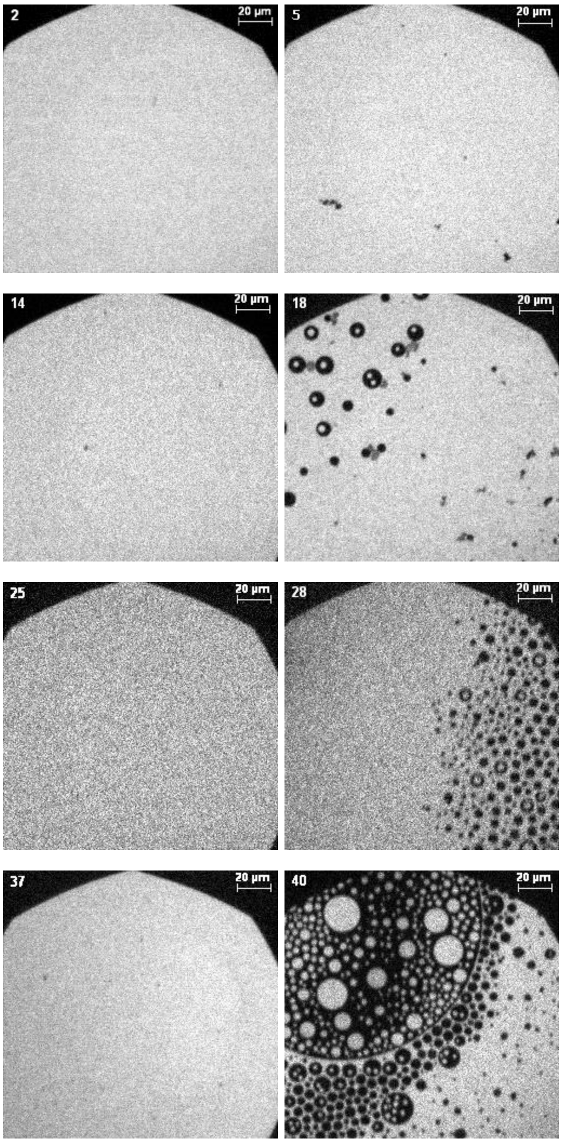

0% cholesterol

10% cholesterol

20% cholesterol

30% cholesterol

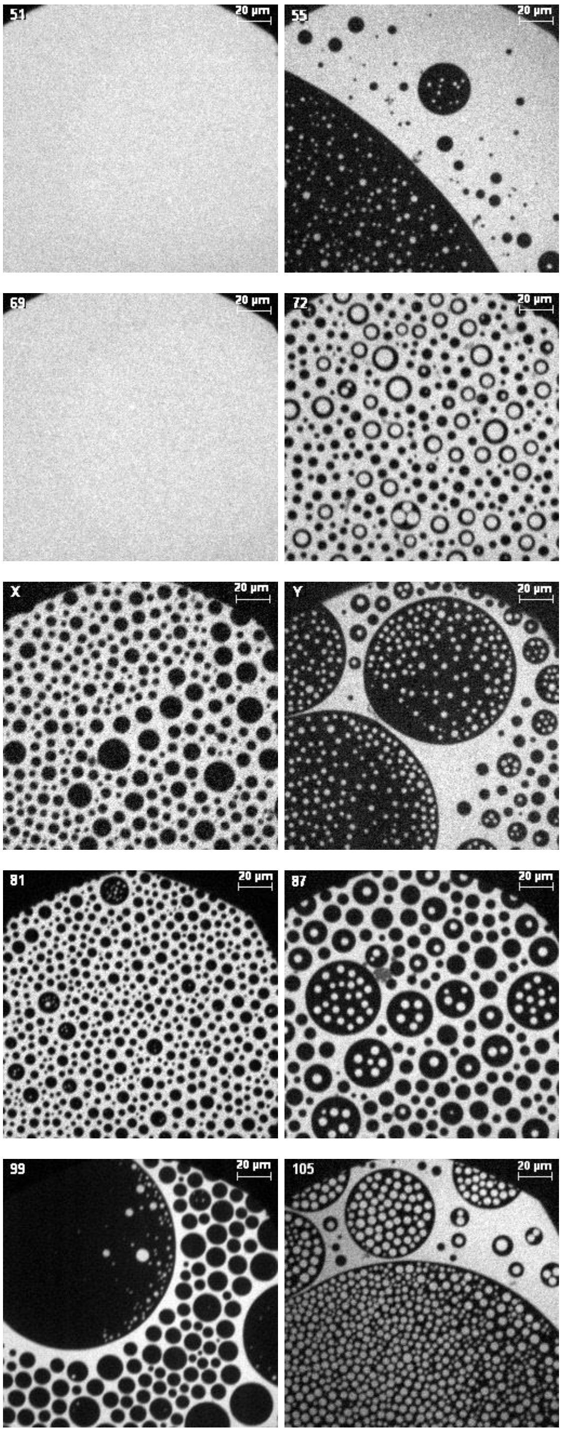

35% cholesterol

40% cholesterol

44% cholesterol

50% cholesterol

60% cholesterol

100% cholesterol

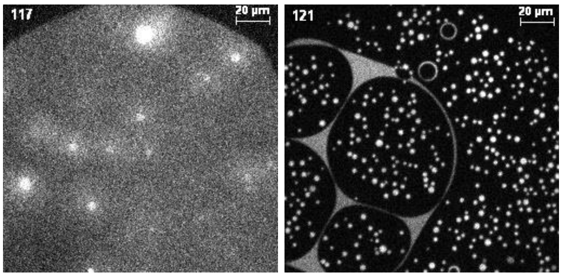

Figure S8. Representative fluorescence microscopy images of lipid monolayers at 10 ± 2 mN/m with different cholesterol content and 0.05 mol% Rh-DHPE. left side: without MBP, right side: with MBP.

S9 Fluorescence microscopy images of all lipid mixtures used, with and without MBP

The respective surface pressures to each image can be found in the table 2 in S10.

0% cholesterol:

Without MBP

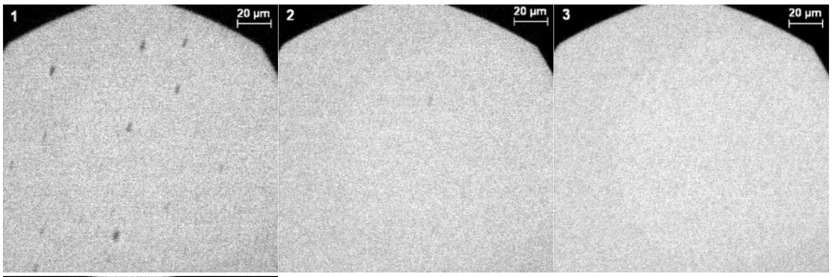

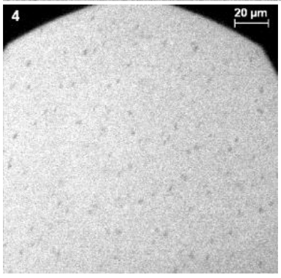

With MBP

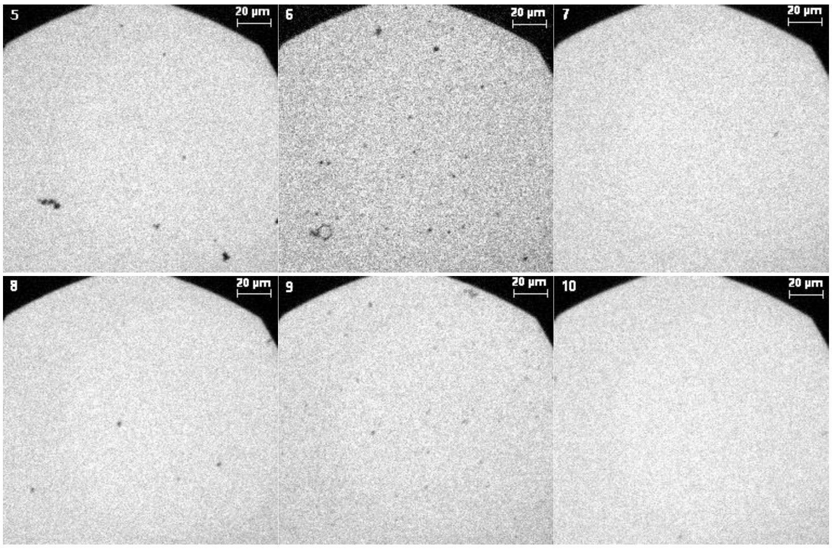

10% cholesterol:

Without MBP

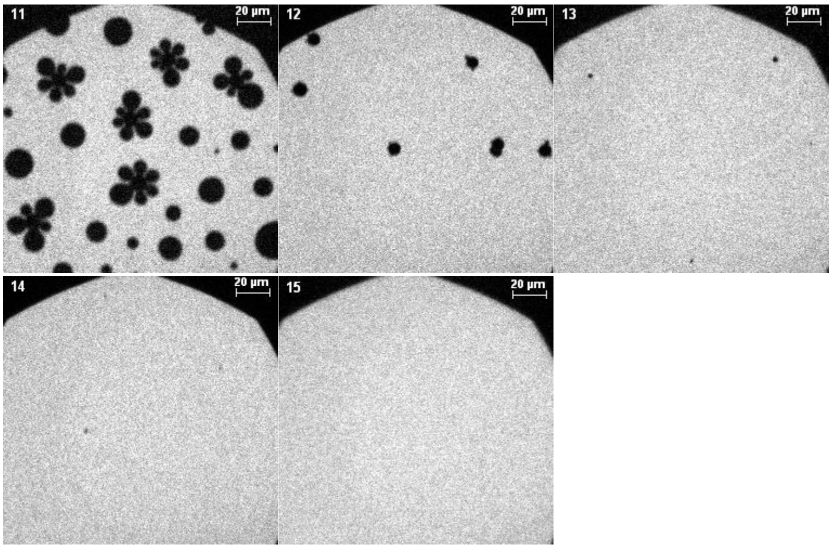

With MBP

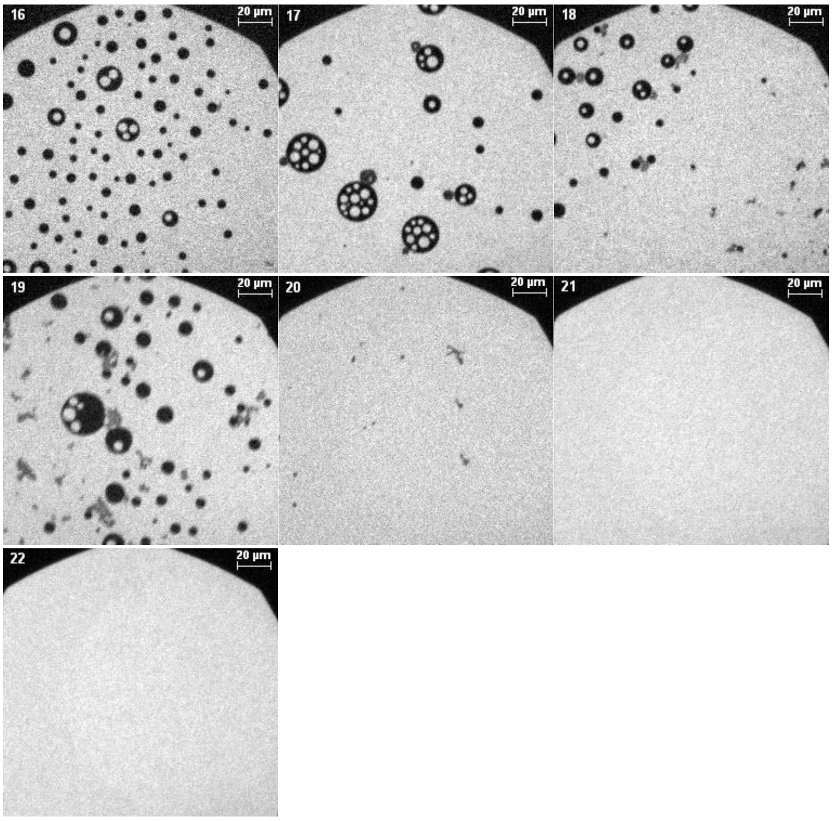

20% cholesterol:

Without MBP

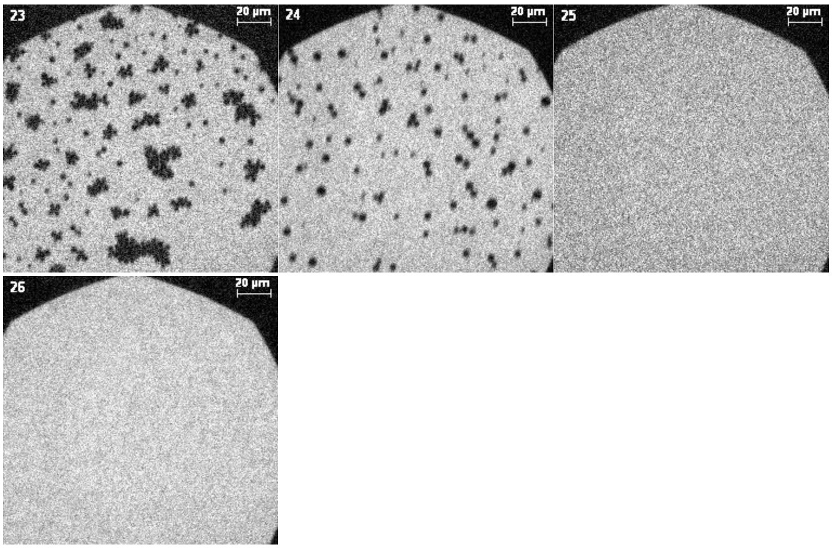

With MBP

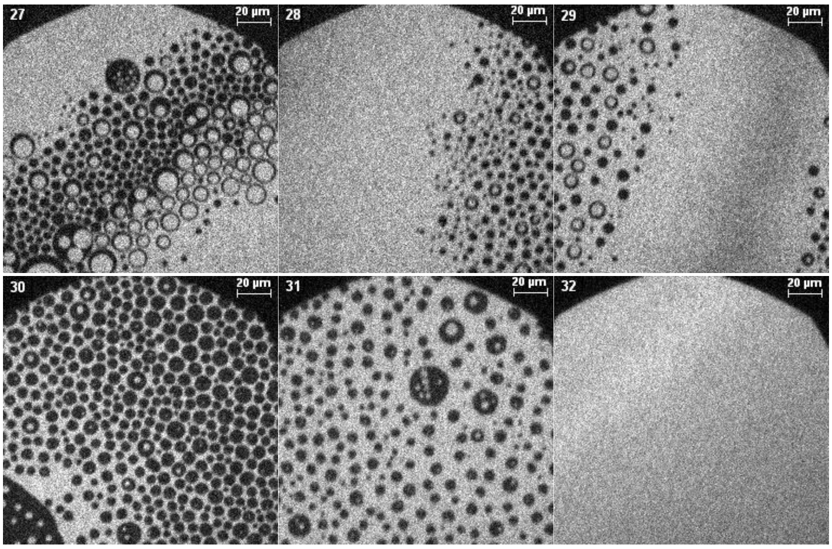

30% cholesterol:

Without MBP

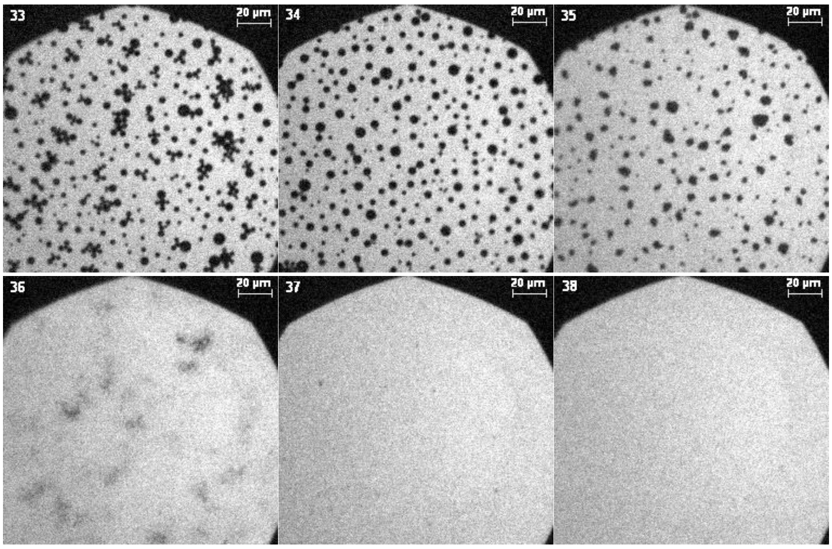

With MBP

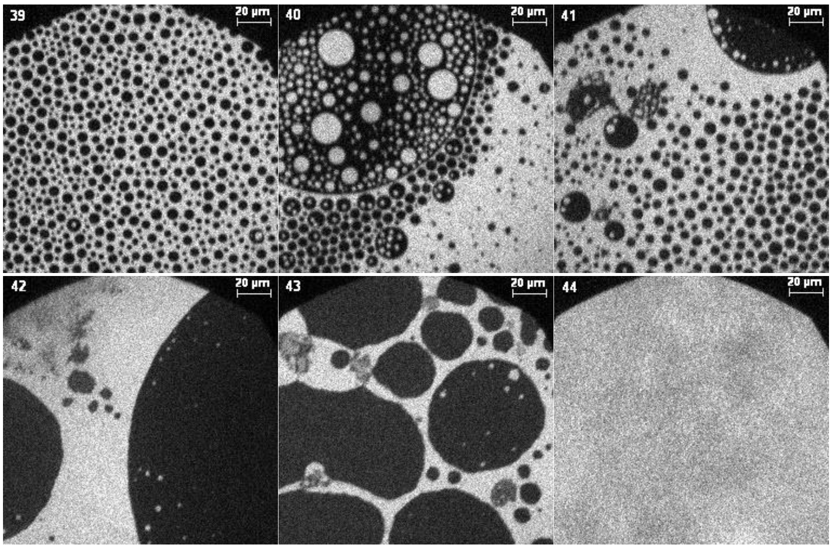

35% cholesterol:

Without MBP

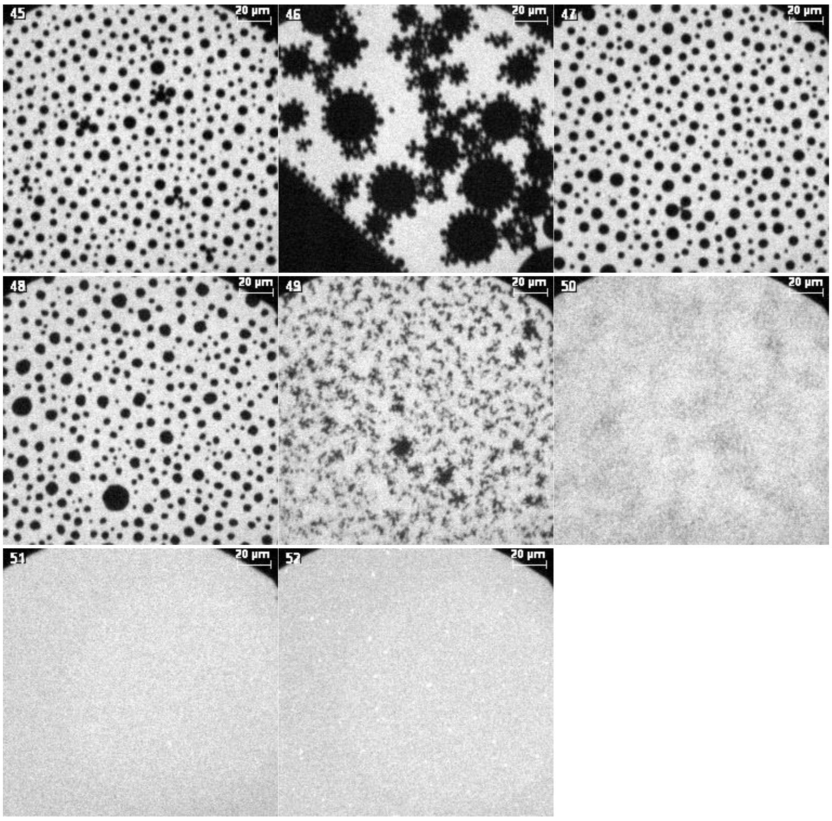

With MBP

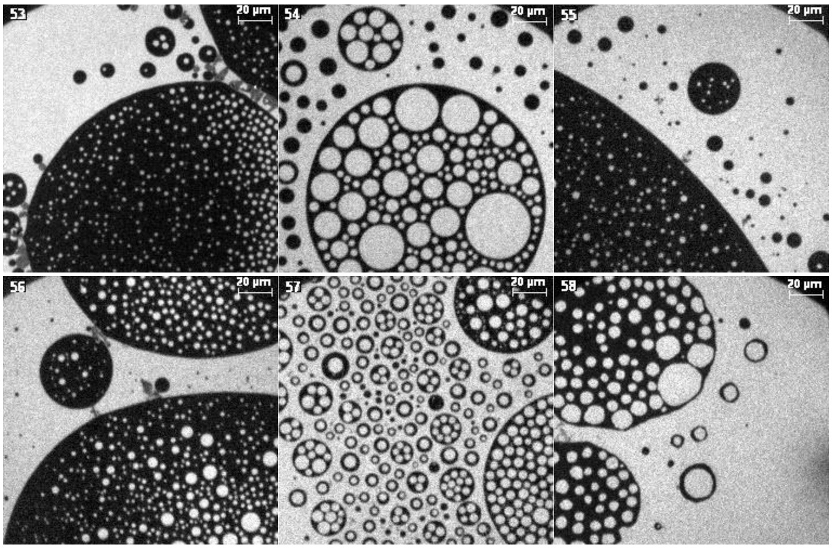

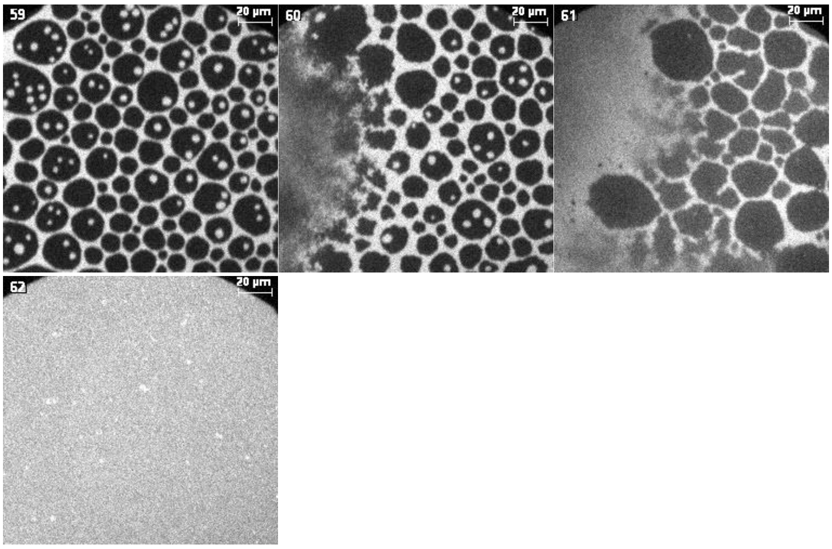

40% cholesterol:

Without MBP

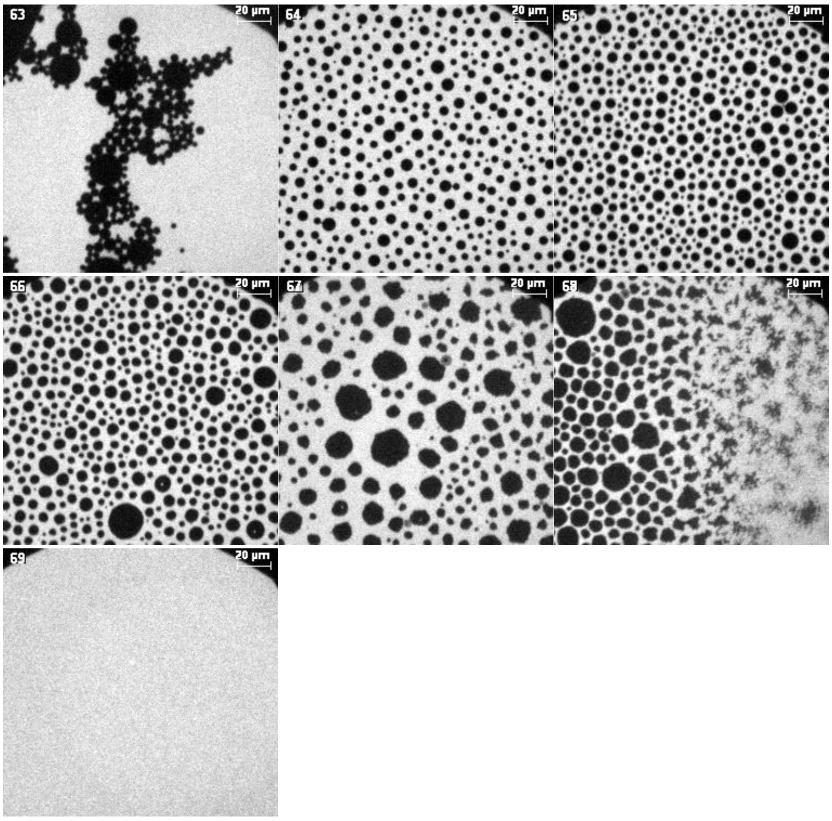

With MBP

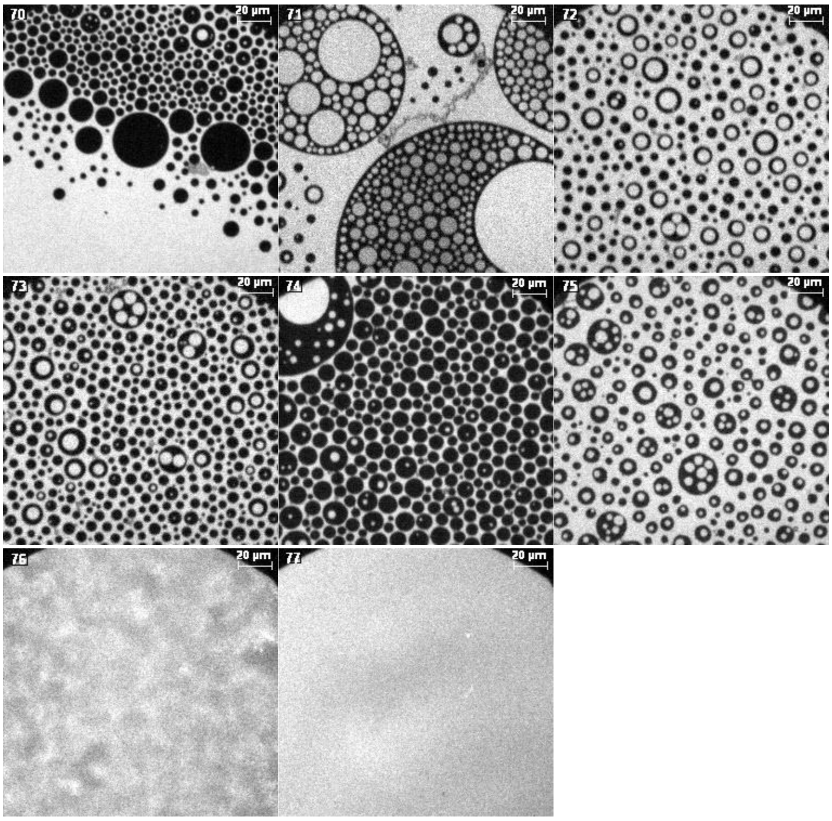

44% cholesterol:

Without MBP

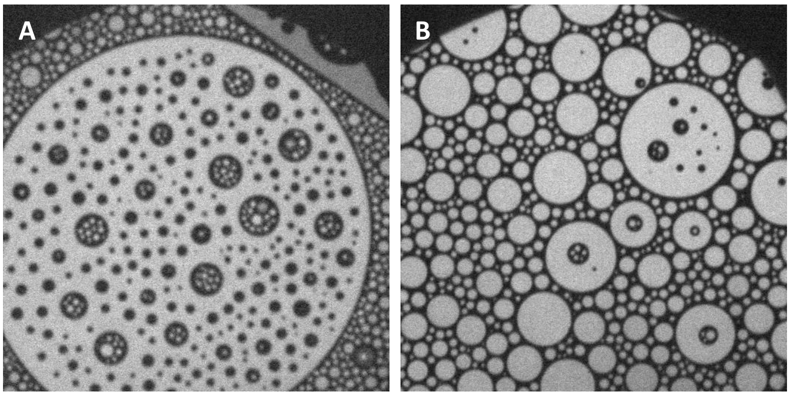

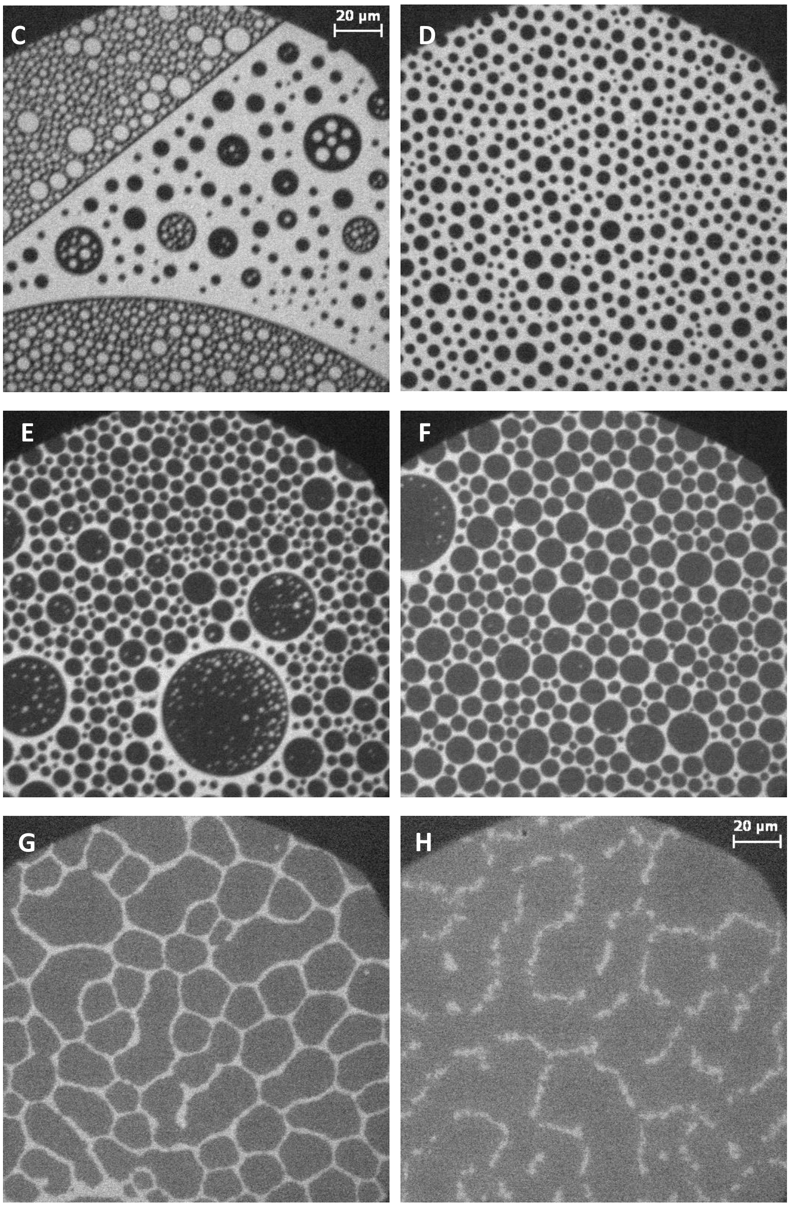
C
20 µm
D
E
F
G
H
20 µm

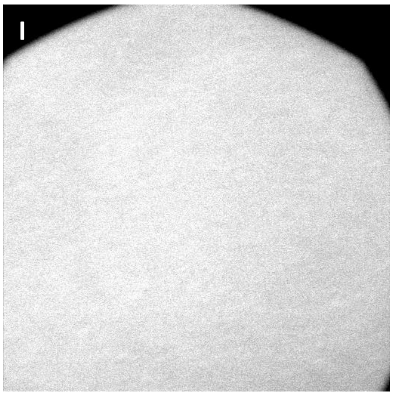

With MBP

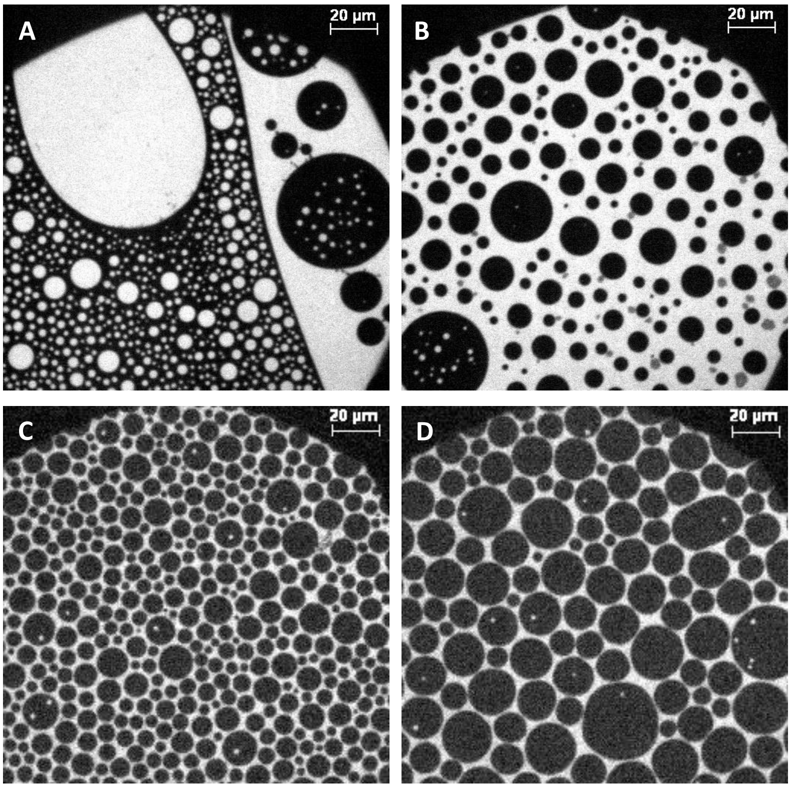

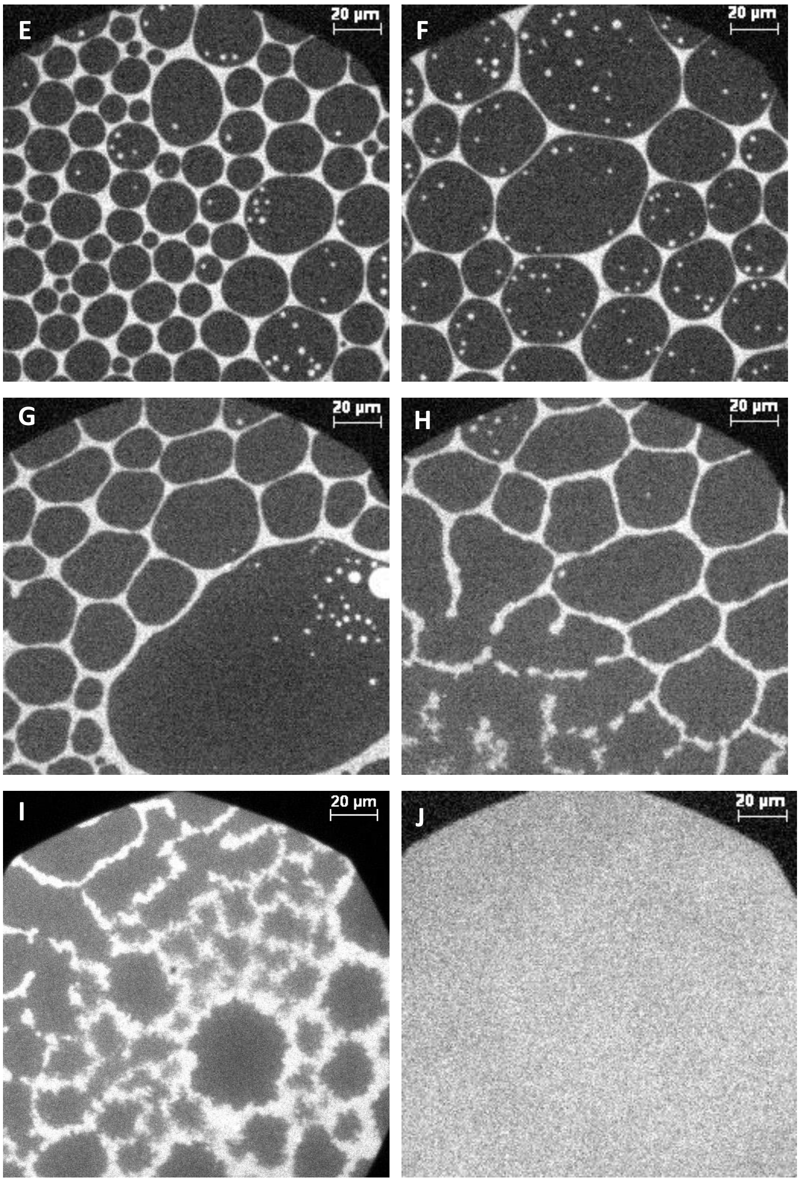
E
20 µm
F
20 µm
G
20 µm
H
20 µm
I
20 µm
J
20 µm

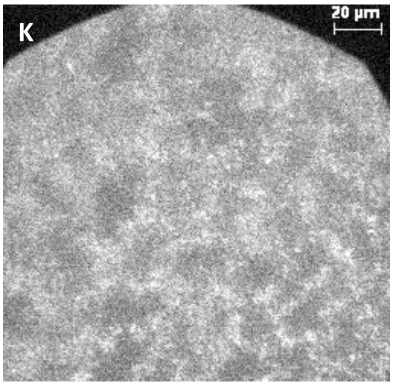

50% cholesterol:

Without MBP

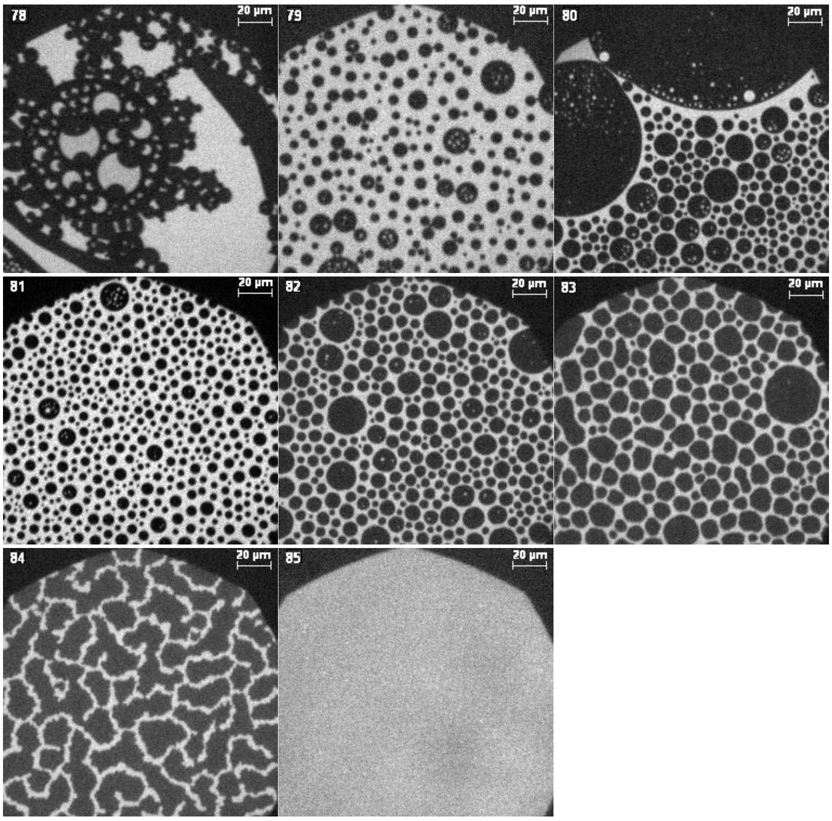

With MBP

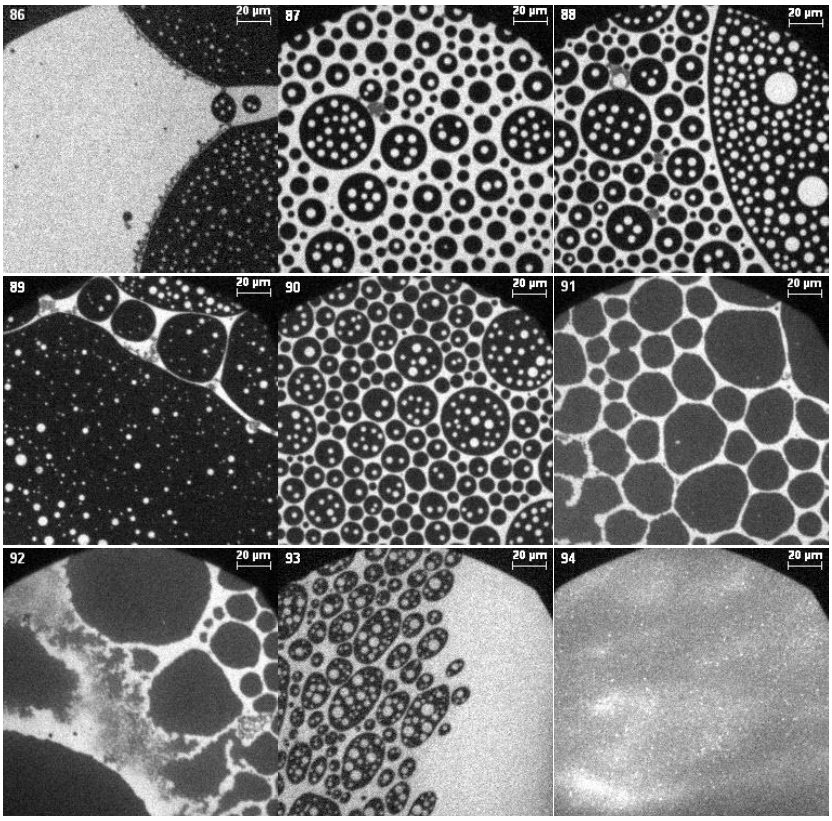

60% cholesterol:

Without MBP

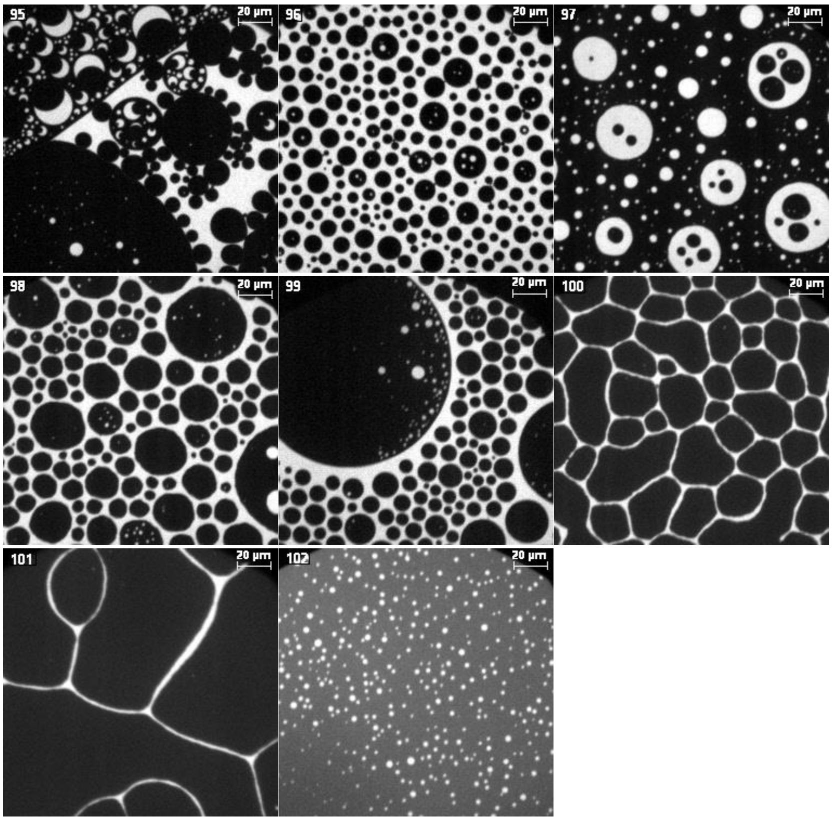

With MBP

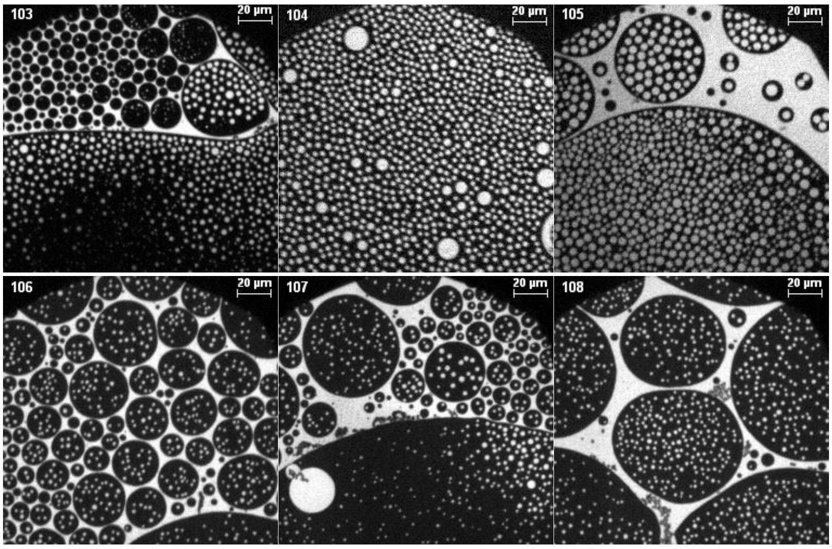

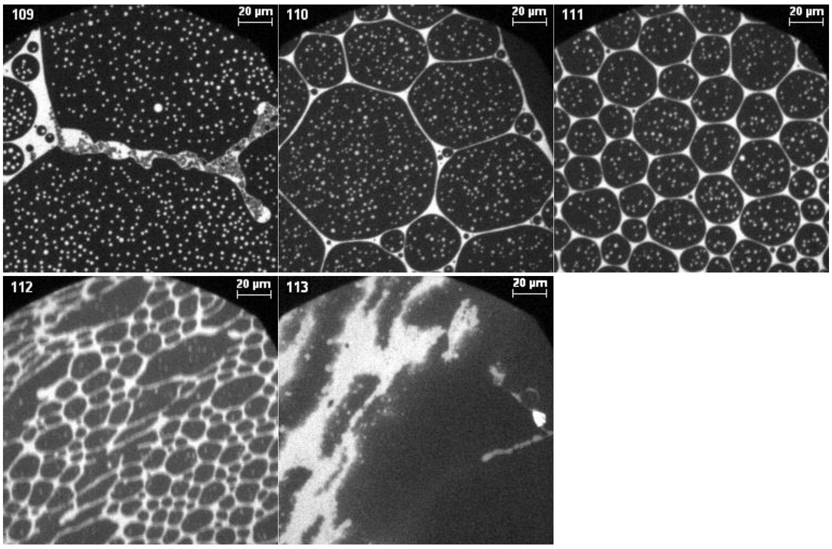

100% cholesterol:

Without MBP

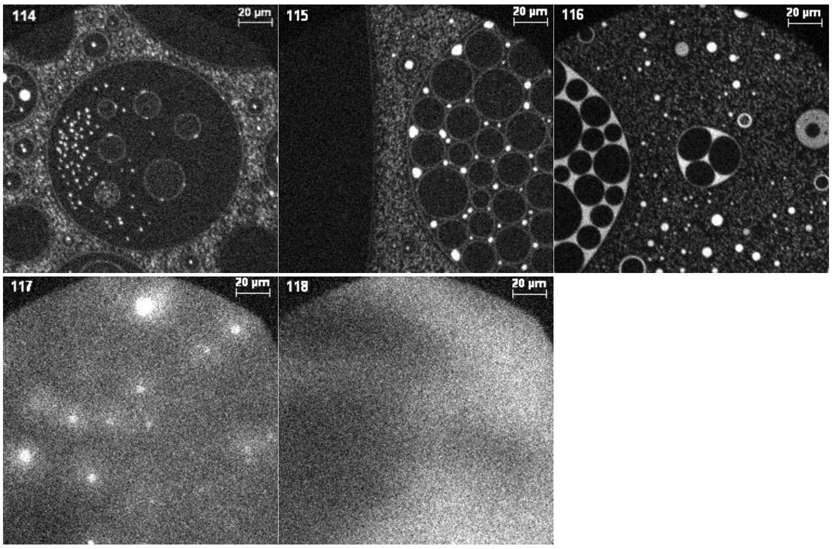

With MBP

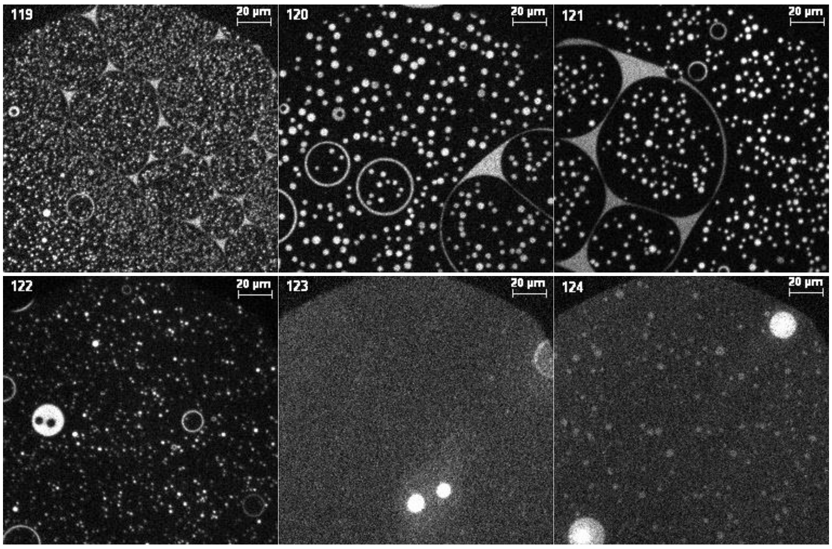

Figure S9. Representative fluorescence microscopy images of the compression of lipid monolayers with different cholesterol content, with and without MBP, and 0.05 mol% Rh-DHPE. The respective surface pressures to each image can be found in the table 2 in S10.

S10 Fluorescence microscopy images with 20x magnification objective

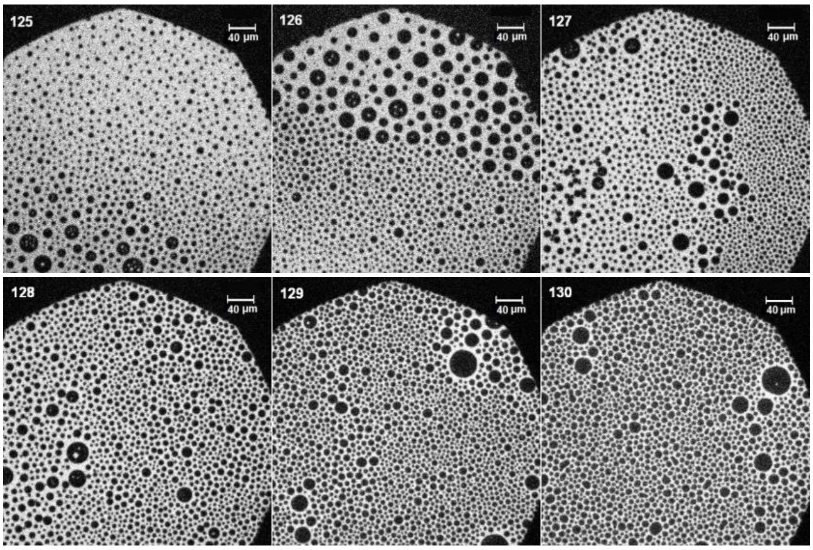

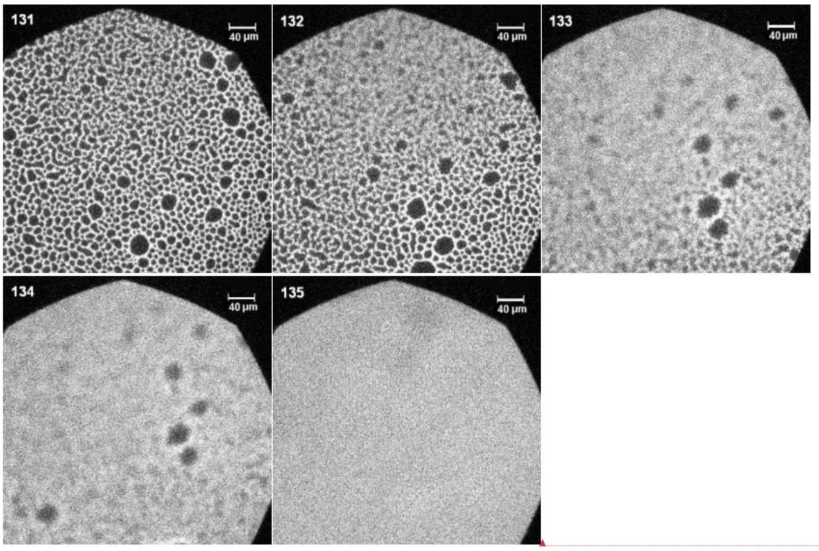

Figure S10. Representative fluorescence microscopy images of lipid monolayer with 44 % cholesterol content and 0.05 mol% Rh-DHPE with a 20x magnification.

Formatted: Not Highlight

S11 Table of surface pressures for all fluorescence images

Table S11. Listing of all fluorescence microscopy images with associated surface pressure.

Deleted: 2

**Cholesterol content in %**	**Image number and associated surface pressure in mN/m without MBP**	**Image number and associated surface pressure in mN/m with MBP**	**Cholesterol content in %**	**Image number and associated surface pressure in mN/m without MBP**	**Image number and associated surface pressure in mN/m with MBP**
0	1 → 0.5<br>2 → 10<br>3 → 30<br>4 → 40	5 → 10<br>6 → 13<br>7 → 16<br>8 → 25<br>9 → 31<br>10 → 41	40	63 → 0<br>64 → 0<br>65 → 1.6<br>66 → 5.4<br>67 → 7<br>68 → 7.8<br>69 → 12	70 → 3<br>71 → 6<br>72 → 11<br>73 → 13.5<br>74 → 23<br>75 → 25<br>76 → 32<br>77 → 39
10	11 → 0<br>12 → 0.2<br>13 → 4<br>14 → 11<br>15 → 36	16 → 5.9<br>17 → 7.6<br>18 → 11<br>19 → 23<br>20 → 25<br>21 → 29<br>22 → 40	44	Figure 4<br>A → 0<br>B → 0<br>C → 5<br>X → 9<br>D → 21<br>E → 25<br>F → 30<br>G → 35<br>H → 36<br>I → 37	Figure 5<br>A → 5<br>B → 5<br>Y → 8.8<br>C → 20.8<br>D → 22<br>E → 25<br>F → 25.8<br>G → 28.7<br>H → 28.9<br>I → 30<br>J → 32<br>K → 37
20	23 → 0.2<br>24 → 1<br>25 → 10.6<br>26 → 36	27 → 9<br>28 → 13<br>29 → 25<br>30 → 30<br>31 → 31<br>32 → 40	50	78 → 0<br>79 → 0<br>80 → 5.6<br>81 → 10<br>82 → 13<br>83 → 14<br>84 → 14.5<br>85 → 19	86 → 4.5<br>87 → 10.8<br>88 → 12<br>89 → 23<br>90 → 24<br>91 → 27<br>92 → 28<br>93 → 29<br>94 → 46
30	33 → 0<br>34 → 0.7<br>35 → 6<br>36 → 6.4<br>37 → 9<br>38 → 17	39 → 7.4<br>40 → 9<br>41 → 16<br>42 → 25<br>43 → 25.4<br>44 → 30	60	95 → 0<br>96 → 0.1<br>97 → 4<br>98 → 5<br>99 → 10<br>100 → 11<br>101 → 35<br>102 → 44	103 → 5<br>104 → 5.3<br>105 → 11<br>106 → 24<br>107 → 24.6<br>108 → 25<br>109 → 25.6<br>110 → 27<br>111 → 27.6<br>112 → 33<br>113 → 34
35	45 → 0<br>46 → 0<br>47 → 3.5	53 → 7.2<br>54 → 8.8<br>55 → 10	100	114 → 0<br>115 → 0<br>116 → 0.4	119 → 6.2<br>120 → 6.6<br>121 → 10

	48 → 4.4<br>49 → 4.8<br>50 → 5<br>51 → 9<br>52 → 44	56 → 11.7<br>57 → 16<br>58 → 23<br>59 → 23.1<br>60 → 23.4<br>61 → 30<br>62 → 32		117 → 10<br>118 → 26	122 → 22<br>123 → 33<br>124 → 45
44 with 20x magnification	125 → 6.2<br>126 → 14.6<br>127 → 23.3<br>128 → 27.1<br>129 → 32.4<br>130 → 34<br>131 → 34.5<br>132 → 34.9<br>133 → 35<br>134 → 35.5<br>135 → 38				
